# Absence of YhdP, TamB, and YdbH leads to defects in glycerophospholipid transport and cell morphology in Gram-negative bacteria

**DOI:** 10.1371/journal.pgen.1010096

**Published:** 2022-02-28

**Authors:** Martin V. Douglass, Amanda B. McLean, M. Stephen Trent

**Affiliations:** 1 Department of Infectious Diseases, College of Veterinary Medicine, University of Georgia, Athens, Georgia, United States of America; 2 Department of Microbiology, College of Arts and Sciences, University of Georgia, Athens, Georgia, United States of America; Max Planck Institute for Terrestrial Microbiology: Max-Planck-Institut fur terrestrische Mikrobiologie, GERMANY

## Abstract

The outer membrane (OM) of Gram-negative bacteria provides the cell with a formidable barrier that excludes external threats. The two major constituents of this asymmetric barrier are lipopolysaccharide (LPS) found in the outer leaflet, and glycerophospholipids (GPLs) in the inner leaflet. Maintaining the asymmetric nature and balance of LPS to GPLs in the OM is critical for bacterial viability. The biosynthetic pathways of LPS and GPLs are well characterized, but unlike LPS transport, how GPLs are translocated to the OM remains enigmatic. Understanding this aspect of cell envelope biology could provide a foundation for new antibacterial therapies. Here, we report that YhdP and its homologues, TamB and YdbH, members of the “AsmA-like” family, are critical for OM integrity and necessary for proper GPL transport to the OM. The absence of the two largest AsmA-like proteins (YhdP and TamB) leads to cell lysis and antibiotic sensitivity, phenotypes that are rescued by reducing LPS synthesis. We also find that *yhdP*, *tamB* double mutants shed excess LPS through outer membrane vesicles, presumably to maintain OM homeostasis when normal anterograde GPL transport is disrupted. Moreover, a *yhdP*, *tamB*, *ydbH* triple mutant is synthetically lethal, but if GPL transport is partially restored by overexpression of YhdP, the cell shape adjusts to accommodate increased membrane content as the cell accumulates GPLs in the IM. Our results therefore suggest a model in which “AsmA-like” proteins transport GPLs to the OM, and when hindered, changes in cell shape and shedding of excess LPS aids in maintaining OM asymmetry.

## Author summary

Much like armor, the OM of Gram-negative bacteria serves as the cell’s first line of defense against harsh environments and toxic molecules. The two major components of the OM are LPS and GPLs. To offer effective protection, the cell must maintain the appropriate balance of LPS and GPLs at the bacterial surface. Here we report that members of the “AsmA-like” family, YhdP, TamB, and YdbH contribute to the OM barrier by aiding in GPL transport and are critical for antibiotic resistance.

## Introduction

**Fig 1:**
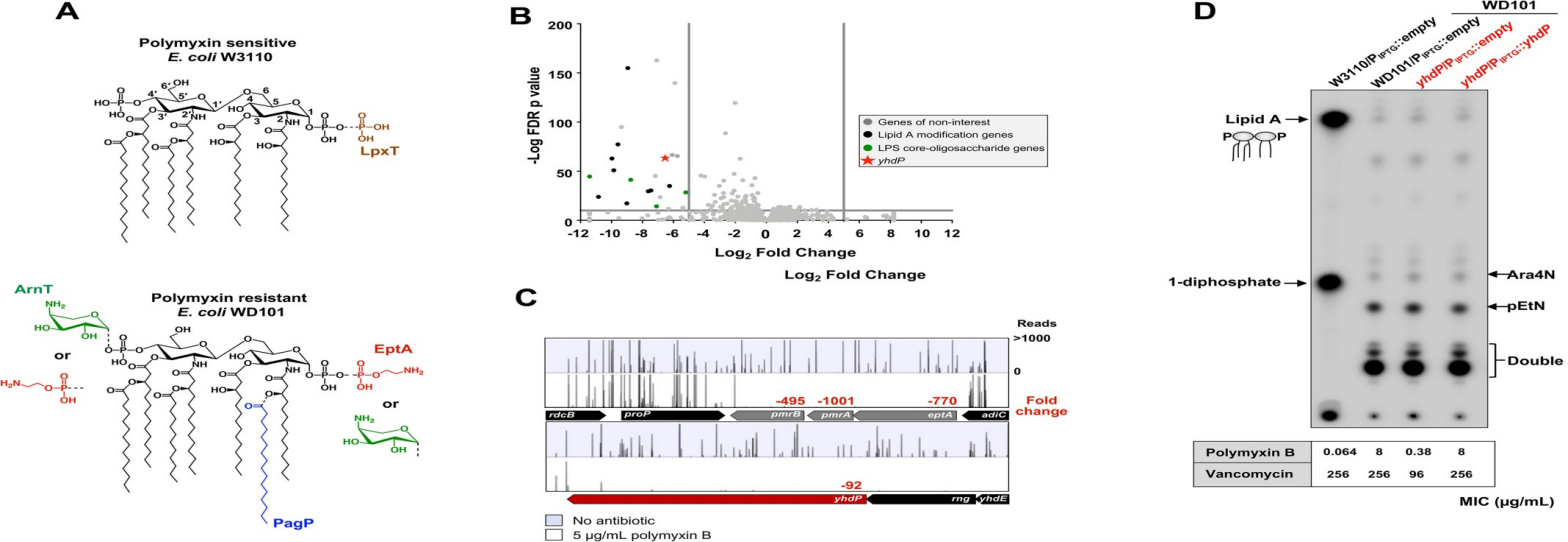
Tn-Seq analysis of polymyxin resistant *E*. *coli* identifies *yhdP* to be essential in the presence of polymyxin. (A) In the presence of polymyxin, the PmrA transcriptional regulator increases expression of proteins required for modification of lipid A, such as EptA and ArnT. PmrA is a constitutively active in strain WD101. Top: Chemical structure of wild-type lipid A with additional phosphate added by LpxT in brown. Bottom: Structure of lipid A under conditions in which PmrAB are activated showing pEtN addition by EptA (red) and Ara4N addition by ArnT (green). PmrA activation leads to inhibition of LpxT. Also, an additional acyl chain (C16:0) can also be added by PagP (blue); however, PagP expression is not controlled by PmrAB. (B) Volcano plot of genes revealed by Tn-Seq analysis. Genes expected to be essential in polymyxin resistance have a cut-off of log_2_ fold change < -4 (vertical grey line), and a False Discovery Rate (FDR) p value <0.05 (horizontal grey line). (C) Tn insertion profile of *yhdP*. The sites of Tn insertions were identified by deep sequencing and mapped onto the W3110 reference genome. Shown are Tn insertion profiles of the *pmrA* and *yhdP* loci. The height of each line in the profile represents the number of sequencing reads corresponding to a Tn insertion at the indicated genome position. In red is the fold change of RPKM value of the genes listed in the presence of polymyxin relative to the absence of polymyxin control. A total of three biological replicates where completed. The profile shown is that of a single biological replicate, but the fold change is the average of all replicates. (D) ^32^P-labeled lipid A profiles of *yhdP* mutants. Cultures were grown to mid-log phase at 37°C and 100 μM IPTG was used for plasmid induction. Lipid A was isolated, separated by TLC and visualized by phosphorimaging. Lipid A species are indicated. Polymyxin and vancomycin MIC from E-test are indicated below the TLC. Both TLC and antibiotic sensitivity data are representative of 3 biological experiments.

The cell envelope of Gram-negative bacteria, such as *Escherichia coli*, provides a formidable barrier that protects the cell from toxic molecules under a wide range of environmental conditions [1]. The cell envelope encompasses a symmetrical membrane bilayer known as the inner membrane (IM), an aqueous periplasmic space, and a secondary outer membrane (OM) that surrounds the peptidoglycan layer. Unlike the symmetrical IM that consist of glycerophospholipids (GPLs), the OM is asymmetrical with GPLs found in the inner leaflet and the glycolipid lipopolysaccharide (LPS) located in the outer leaflet [2]. LPS is divided into three major components: the conserved lipid A domain, the core oligosaccharide, and the distal O-antigen [3]. Lipid A is a glucosamine disaccharide that is *bis-*phosphorylated and, in *E*. *coli*, is decorated with 6 acyl chains (**Fig 1A**) [3]. Negatively charged phosphates of LPS facilitate cross-bridging with divalent cations promoting strong lateral interactions between adjacent LPS molecules resulting in a selective permeability barrier [3].

Interestingly, the highly conserved lipid A domain is not static in nature as the sugars and acyl chains are modified so the cell can adapt to a dynamic range of environments [4]. Lipid A modifications also promote resistance to key cationic antimicrobials, such as the polymyxins, the so-called “last-resort” antibiotic in treating Gram-negative infections. Polymyxins are cationic antimicrobial peptides that bind to the negatively-charged phosphate groups of lipid A, which then leads to disruption of cell envelope structure and ultimately to cell death [4]. Many Gram-negatives become resistant to polymyxin by masking lipid A phosphate groups. In *E*. *coli*, the PmrAB two-component system activates the expression of the enzymes ArnT and EptA that catalyze the addition of 4-amino-4-deoxy-L-arabinose (Ara4N) and phosphoethanolamine (pEtN), respectively [3, 4] (**Fig 1A**). The addition of these amine-containing residues leads to polymyxin resistance.

The major components of the Gram-negative OM are synthesized at the cytoplasmic face of the IM. How Gram-negative bacteria transport OM components from their origin of synthesis (IM) while maintaining OM asymmetry has been the subject of intense research. The Lol (localization of lipoproteins) transport apparatus system is responsible for ferrying lipoproteins to the OM from the IM [1]. Whereas the intermembrane transport of LPS is accomplished by the 7 member translocation machine, Lpt (LPS transport) [1]. However, the transport of GPLs from the IM to the OM remains enigmatic. Bioinformatic approaches have identified a common domain that is found in eukaryotic GPL transporters in the Gram-negative bacterial proteins TamB and AsmA [5]. Furthermore, YhdP, a homologue of TamB and AsmA, was proposed to facilitate GPL transport from the IM to the OM, although the loss of this potential GPL transporter is not lethal to the cell nor was the GPL membrane content directly measured [6].

To identify novel genes that participate in polymyxin resistance and OM integrity, we performed saturated transposon (Tn) mutagenesis in an *E*. *coli* K-12 strain in which the PmrA transcriptional regulator is constitutively active (WD101), screening for genes critical for survival in sublethal doses of the OM targeting drug polymyxin. Here we identify several genes of interest, including *yhdP*, to be essential in polymyxin resistance. We focused initially on YhdP’s role in the cell as it was previously described by Mitchell and colleagues to be important for maintaining the OM permeability barrier [7, 8] and its potential role in anterograde GPL transport [6].

Our findings here bolster the previous evidence that YhdP and its homologues, TamB and YdbH, aid in GPL transport. We reveal that absence of YhdP and TamB leads to excess amounts of outer membrane vesicles (OMVs) that are rich in LPS, presumably to compensate for the lack of proper GPL transport to the OM. Furthermore, in the absence of YhdP, TamB, and a third AmsA-like protein, YdbH, the cell is not viable but can be partially rescued by overexpression of YhdP. Notably, in this background, GPLs in the IM begin to accumulate. Overall, we propose a model where YhdP and its homologues promote anterograde GPL transport, and absence of these GPL transporters lead to the release of LPS through OMVs to maintain appropriate GPL to LPS ratios in the OM.

## Results

### Loss of YhdP is lethal in the presence of polymyxin

To gain further insight into additional mechanisms promoting polymyxin resistance and OM integrity, we used a Tn-seq approach that pairs random transposon (Tn) insertion library construction with Next-Gen sequencing. We constructed an *E*. *coli* Tn insertion library in WD101, derived from W3110, that contains mutations in the *pmrA* gene resulting in a PmrA constitutive phenotype. WD101 is highly resistant to polymyxin antibiotics with heavily modified lipid A (**Fig 1A**) [9]. Triplicate Tn libraries consisting of ~450,000 mutants per replicate were challenged with 0 μg/mL of polymyxin or a sublethal dose of 5 μg/mL of polymyxin until mid-log phase was reached [10, 11]. As expected, genes involved in lipid A modification did not accumulate Tn insertions in the presence of polymyxin (**Fig 1B and 1C**). For example, it was expected that *eptA/pmrA/pmrB* would be critical for fitness in the presence of polymyxin, but dispensable in the absence of antibiotic (**Fig 1C**). Compared to the no antibiotic control, *pmrB*, *pmrA* and *eptA* had -495, -1001, and -770-fold fewer Tn insertions when challenged with polymyxin, respectively, validating the screen (**Fig 1C**). Mutations impacting the core oligosaccharide of LPS were also important for fitness in the presence of polymyxin (**Fig 1B**) as truncation of the core increases antibiotic susceptibility in general, as seen against colistin (polymyxin E), rifampicin, and erythromycin [12].

**Fig 2:**
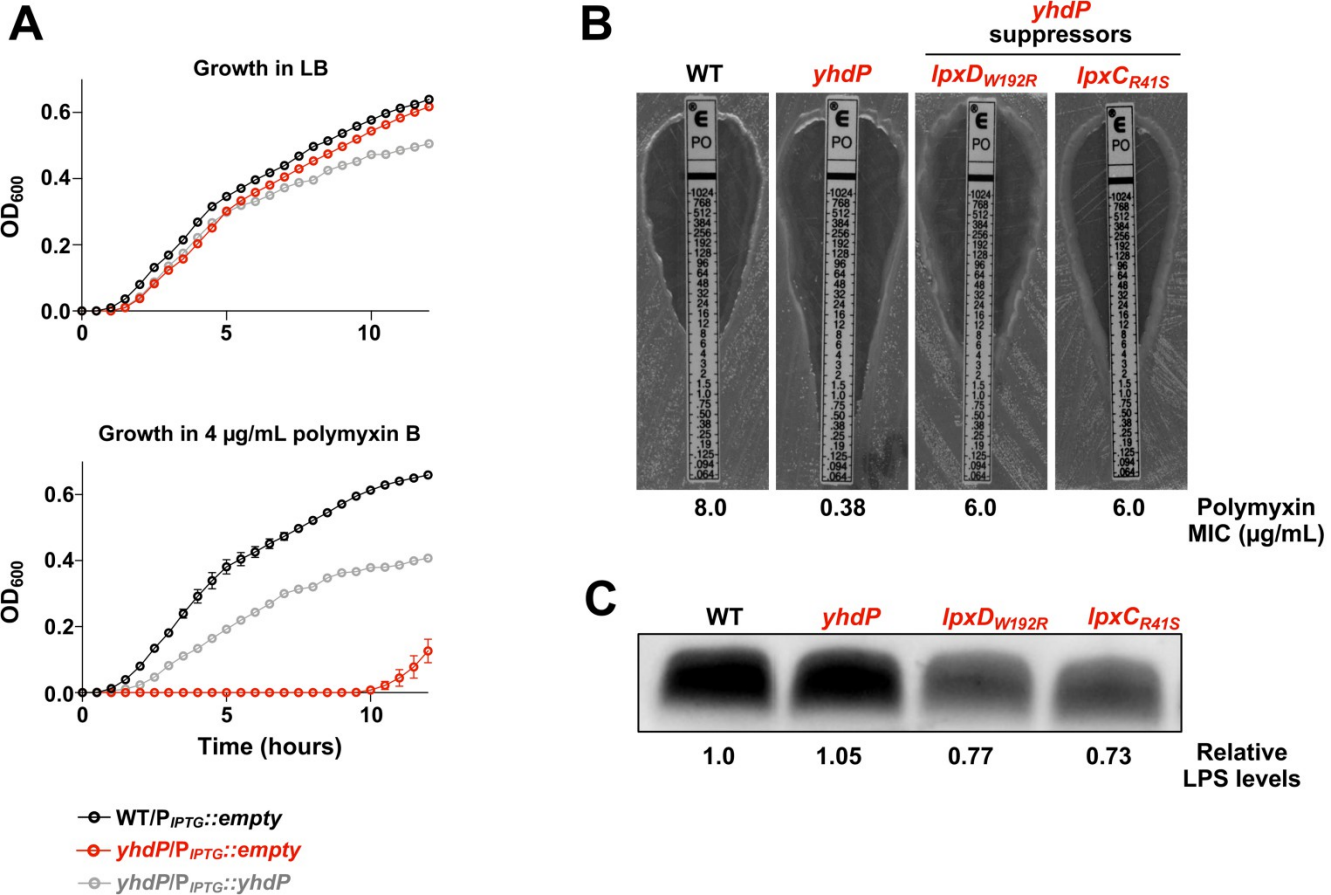
WD101 *yhdP* polymyxin sensitivity is restored by reduction in LPS levels. (A) Growth curve of WD101 *yhdP* in the presence of 0 or 4 μg/mL polymyxin. Strains were monitored by OD_600_ every 30 minutes at 37°C and 100 μM IPTG was used for plasmid induction. Error bars represent standard deviation (SD) from technical triplicates (data representative of 3 biological replicates). (B) Polymyxin MIC of *yhdP* suppressors. Mid-log phase cultures were used for E-test at 37°C overnight. MIC is indicated where zone of clearance meets marked number on E-test. **C**. LPS levels of *yhdP* suppressors. LPS levels were determined by staining of LPS following SDS-PAGE of proteinase K-treated whole cell lysates of indicated strains. Polymyxin MIC and LPS levels are representative of 3 biological experiments.

However, we found the gene *yhdP* to be critical for fitness in the presence of polymyxin, with a fold change of -92 (**Fig 1C**). *yhdP* has not been previously associated with resistance to cationic antimicrobial peptides in *E*. *coli*. To test the impact of *yhdP* on fitness, we constructed single mutants lacking *yhdP* in the WD101 background. In the absence of antibiotic, deletion of *yhdP* did not affect growth (**Fig 2A**); however, in the presence of 4 μg/mL of polymyxin, the *yhdP* mutant had a severe growth defect compared to the parent (**Fig 2A**). Polymyxin resistance could be rescued, at least partially, by plasmid complementation. Similar results were seen when determining the minimum inhibitory concentration (MIC) of polymyxin by Epsilometer-test (E-test). The parent WD101 showed a MIC of 8 μg/mL whereas loss of *yhdP* reduced the MIC to 0.38 μg/mL (**Fig 2B**). The MIC for *yhdP* was fully restored when *yhdP* was expressed *in trans*. Overall, our Tn-seq results correctly identified *yhdP* to be essential for polymyxin resistance in *E*. *coli*.

Current understanding of Gram-negative bacterial resistance to polymyxin is primarily due to the bacterium’s ability to decrease the negative charge of its surface. In *E*. *coli*, this occurs by the addition of Ara4N and pEtN to the phosphate groups of lipid A (**Fig 1A**) [3]. With the deletion of *yhdP* leading to polymyxin sensitivity similar to *E*. *coli* lacking LPS modifications, we questioned whether YhdP influences Ara4N or pEtN addition to lipid A. Surprisingly, TLC analysis of the lipid A fraction from ^32^P-labeled bacteria showed no changes in the lipid A profile in the *yhdP* mutant compared to wild-type (WT) WD101 (**Fig 1D**). Overexpression of *yhdP* also had no effect on lipid A structure (**Fig 1D**). Polymyxin-sensitive strain W3110 produced the characteristic hexa-acylated lipid A species, one that is *bis*-phosphorylated and a second species (~1/3^rd^ of the lipid A fraction) that has an additional phosphate at the 1-position added by LpxT (**Fig 1A and 1D**) [13]. The WD101 parent, as well as the mutant strains, all produced highly modified lipid A resulting in slower migrating species with either a single or both phosphate groups modified [13]. This data suggests that *yhdP* essentiality in polymyxin resistance is independent of lipid A modifications and is dominant over PmrA activity.

### Suppressors in lipid A synthesis rescue polymyxin resistance in the absence of *yhdP*

YhdP is an IM protein with 1266 amino acids that was initially reported to be important for maintenance of OM permeability [7, 8]. We began by determining if previous published suppressors for *yhdP’s* phenotypes rescues our WD101 *yhdP* polymyxin sensitivity. Previous reports found that absence of *yhdP* increases sensitivity to vancomycin, an antibiotic that normally doesn’t penetrate the Gram-negative OM, and increased sensitivity to SDS-EDTA (sodium dodecyl sulfate in combination with ethylenediaminetetraacetic acid) that leads to OM disruption. In either case, resistance is restored when synthesis of Enterobacterial Common Antigen (ECA), a conserved glycoform in *Enterobacteriaceae*, is halted [8]. The role of ECA still remains unclear, but three forms are present in the cell: LPS-linked ECA [14], phosphatidylglycerol-linked ECA [15], and cyclic ECA that remains in the periplasm [16]. Specifically, it was reported that deletion of *wzzE*, a protein that controls ECA chain length and is responsible for the presence of cyclic ECA, restored vancomycin and SDS-EDTA resistance in the absence of *yhdP* [8]. For WD101 *yhdP*, we also found that vancomycin resistance is restored upon deletion of *wzzE* by performing efficiency of plating (EOP) assays (**S1A Fig**). Yet, absence of *wzzE* does not rescue polymyxin resistance in the *yhdP* mutant, nor does it alter resistance of WT WD101 (**S1A Fig**). Since previously reported suppressors for *yhdP* do not rescue our phenotype, we screened for WD101 *yhdP* suppressors that were able to grow on plates containing 10 μg/mL of polymyxin. Surviving colonies were isolated and their mutations mapped by whole genome sequencing. We found multiple suppressors in genes involved the early steps of lipid A biosynthesis, specifically in *lpxC* and *lpxD* (**Figs 2B and S1B**). Interestingly, not only do these suppressor mutations rescue polymyxin resistance, the suppressors also increase vancomycin resistance, suggesting an improvement in OM asymmetry (**S1B Fig**).

Mutations in lipid A synthesis typically result in decreased vancomycin resistance due to lowered production of LPS, causing impaired OM asymmetry. A classic example are strains harboring the mutation *lpxC101*, an allele resulting in decreased activity of LpxC, which catalyzes the first committed step of lipid A synthesis [17]. To determine the effect of our *yhdP* suppressors on LPS levels, we measured LPS levels of whole cells in our *yhdP* mutant strains (**Fig 2C**). We found that absence of *yhdP* does not affect LPS levels, similar to what was reported after deletion of *yhdP* in a polymyxin-sensitive K-12 strain [6]. However, suppressors WD101 *yhdP lpxD*_*W192R*_ and WD101 *yhdP lpxC*_*R41S*_ both show a ~30% decrease in LPS levels compared to the parent strain (**Fig 2C**). Therefore, decreasing LPS levels restores polymyxin resistance, and against the canon, restores vancomycin resistance (**S1B Fig**).

### YhdP homologue, TamB, is essential in WD101 *yhdP*

**Fig 3:**
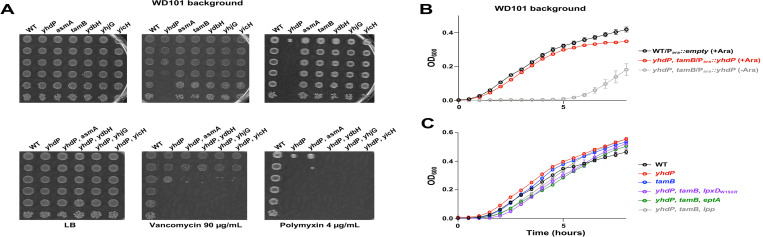
Impact on growth and antibiotic resistance in strains lacking AsmA-like proteins. (A) YhdP homologues are not essential for polymyxin or vancomycin resistance in WD101. Serial dilutions of the indicated strains were spotted on LB plates containing either polymyxin or vancomycin and grown at 37°C. (B) Loss of *yhdP* and *tamB* in polymyxin resistant WD101 is synthetically lethal. The strain *yhdP*, *tamB/*P_*ara*_::*yhdP* was grown under inducing conditions with 0.2% arabinose (+Ara, red) or under repressing conditions with 0.2% glucose (-Ara, grey) at 37°C. (C) Lowering LPS levels or lipid A modifications allows for deletion of both *yhdP* and *tamB* in WD101. The indicated strains were grown at 37°C. Error bars represent SD from technical triplicates. Data is representative of 3 biological replicates.

YhdP belongs to the AsmA-like family consisting of 5 other inner membrane proteins: AsmA, TamB, YdbH, YicH, and YhjG. Although *yhdP* was the only AsmA-like gene that demonstrated large fold changes in our polymyxin Tn-seq screen, we explored if additional AsmA-like family members are important for antibiotic resistance (**Fig 3A**). In line with the Tn-seq data, individual deletion of the other AsmA-like encoding genes did not affect polymyxin or vancomycin resistance by EOP assay (**Fig 3A**). We next investigated if there is any redundancy among the AsmA-like family members and constructed *yhdP* double mutants (**Fig 3A**). Deleting *asmA*, *ydbH*, *yhjG*, *or yicH* in a WD101 *yhdP* background leads to a slight decrease in vancomycin resistance compared to the *yhdP* parent (**Fig 3A**). Deleting *ydbH*, *yhjG*, or *yicH* in a WD101 *yhdP* background led to a further decrease in polymyxin resistance, whereas the WD101 *yhdP asmA* mutant led to a subtle yet consistent increase in polymyxin resistance (**Fig 3A**). Interestingly, we are unable to generate a *yhdP*, *tamB* double mutant in WD101 after multiple attempts. To demonstrate that *yhdP*, *tamB* is synthetically lethal in this background, we transformed WD101 *yhdP* with a plasmid carrying *yhdP* under the control of an arabinose inducible promoter, creating the strain WD101 *yhdP/*P_ara_::*yhdP*. The *tamB* deletion was then introduced by phage transduction with *yhdP* expressed in trans, resulting in strain WD101 *yhdP*, *tamB/*P_ara_::*yhdP*. In the presence of arabinose, WD101 *yhdP*, *tamB/*P_ara_::*yhdP* grows similar to WD101, but in the absence of arabinose when *yhdP* is no longer expressed, the strain has a severe growth defect and no growth is detected for the first 5 hours, revealing that a *yhdP*, *tamB* double mutant is synthetically lethal in WD101 (**Fig 3B**).

In our depletion growth curve of WD101 *yhdP*, *tamB/*P_ara_::*yhdP*, slight growth appears after 5 hours, suggesting suppressor mutants are obtainable. To identify suppressors that bypass the WD101 *yhdP*, *tamB* lethality, we subjected WD101 *yhdP* mutants to a large-scale transduction with a *tamB*::kan allele. Surviving transductants were isolated and their mutations mapped by whole genome sequencing. All suppressor mutants contained complete deletion of genes *eptA/pmrA/pmrB*. Such a deletion would result in not only loss of pEtN modification of lipid A by EptA, but also loss of Ara4N addition as the enzymatic machinery for Ara4N addition [3] is under PmrA control. Since pEtN is the primary lipid A modification in *E*. *coli*, we generated a WD101 *yhdP* strain lacking *eptA* and tested if *tamB* can be deleted. WD101 *yhdP*, *eptA* readily accepts a *tamB* deletion and does not exhibit a severe growth defect (**Fig 3C**). Additionally, our suppressor WD101 *yhdP lpxD*_W192R_ readily accepts a *tamB* deletion and grows like wild type (**Fig 3C**). Therefore, WD101 can be absent of both *yhdP*, and *tamB* when LPS levels are lowered, or by abolishing lipid A modifications. Cells with modified lipid A do not readily form OMVs compared to the canonical lipid A, due to the addition of covalent head groups to lipid A possibly inducing less negative curvature of the OM—the driving force of OMV formation [18]. OMV formation is a method used by bacterial cells to remodel the OM and to rid the cell of excess LPS, as seen with *lpp* mutants [19]. Because lowering LPS levels and LPS modifications promote WD101 *yhdP*, *tamB* viability, we speculated that our WD101 *yhdP*, *tamB* mutant contains excess LPS that leads to cell death due to a potential absence of OMV formation. To increase OMV shed, we deleted *lpp* in a WD101 *yhdP* background and tested if a WD101 *yhdP*, *lpp* mutant can readily accept a *tamB* deletion. Not only is a WD101 *yhdP*, *tamB*, *lpp* mutant viable, but it grows similar to WT WD101 (**Fig 3C**).

### *yhdP*, *tamB* double mutants lead to multiple cell envelope defects

**Fig 4:**
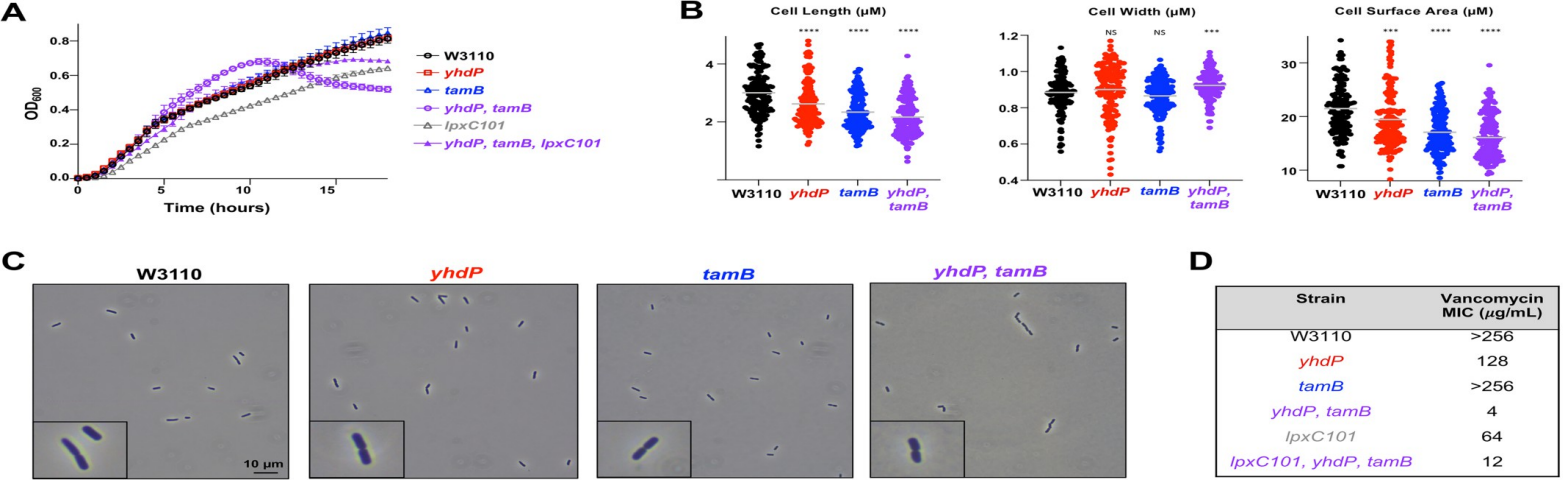
*yhdP*, *tamB* mutants in W3110 exhibit multiple defects associated with the cell envelope. (A) Growth of *yhdP*, *tamB* mutants. Strains were monitored by OD_600_ every 30 minutes at 37°C and error bars represent SD from technical triplicates with data representative of 3 biological replicates. (B) Loss of both *yhdP* and *tamB* results in decreased cell surface area. Cell width and length of listed strains were measured using MicrobeJ software after strains were grown to mid-log phase and imaged at 1000x on agarose pads, cell surface area was calculated using cell width and length. Grey bar indicates mean. T-test used between strains. 0.0001>P****, 0.001>P***. (C) Microscopy of *yhdP* and *tamB* mutants. Phase contrast microscopy (1000x) of the indicated cells with black scale bar set to 10 μm. Image in bottom left corner of dividing cell is a 300x zoom of the larger image. Microscopy data is representative of two biological experiments. (D) The vancomycin MIC of *yhdP*, *tamB* mutants and respective control strains. MICs were determined by E-test after overnight growth at 37°C.

Because a *yhdP*, *tamB* mutant is not viable in the WD101 background unless lipid A modifications are absent or total LPS levels are lowered, we switched to a typical K-12 strain, W3110. W3110 is the parent of WD101, contains a WT *pmrA* allele and does not modify its lipid A under standard laboratory conditions in rich media [9]. As in WD101, single deletions of *yhdP* or *tamB* in W3110 did not result in growth defects (**Fig 4A**), but the double mutant shows a mucoid morphology and undergoes cell lysis during stationary phase (**Fig 4A**). We assessed morphologic changes by phase contrast microscopy and quantified changes in cell width, length, and surface area using MicrobeJ with at least 10 fields of view at 100x magnification on >150 cells. The *yhdP* and *tamB* single mutants show a decrease in cell length but no significant changes in cell width, resulting in a minor reduction in cell surface area (**Fig 4B and 4C**). The double mutant, *yhdP*, *tamB* resulted in a more visible rounding of the cell leading to increased cell width, decreased cell length, and a significant decrease in cell surface area. As recently shown by Oldewurtel et al. [20], this data supports an overall reduction in cell dry-mass (**Fig 4B and 4C**) [20]. Because *yhdP* is necessary for complete vancomycin resistance, we were interested if loss of *tamB* exacerbates vancomycin sensitivity in a *yhdP* background [8]. Deletion of *tamB* does not lead to vancomycin sensitivity in W3110, but a *yhdP*, *tamB* double mutant has a severe sensitivity to vancomycin with a MIC of 4 μg/mL, 32-fold lower than a single *yhdP* mutant (**Fig 4D**) and >64-fold lower than WT W3110. Thus, the OM is severely compromised in a *yhdP*, *tamB* double mutant.

Reducing LPS levels rescued WD101 *yhdP*, *tamB* synthetical lethality and WD101 *yhdP* polymyxin and vancomycin sensitivity, thus we were curious if reducing LPS levels through *lpxC101* could rescue stationary cell lysis and vancomycin sensitivity in W3110 *yhdP*, *tamB*. Although cells harboring the *lpxC101* allele have a growth defect, moving this mutation into a *yhdP*, *tamB* background rescued stationary cell lysis (**Fig 4A**) and increased growth rate. Lowering of LPS normally leads to a decrease in vancomycin resistance [17], which we observe with our *lpxC101* mutant in WT W3110 (**Fig 4D**). However, introduction of the *lpxC101* allele into *yhdP*, *tamB* results in a 3-fold increase in vancomycin resistance (MIC of 12 μg/mL) (**Fig 4D**), suggesting that reduced LPS levels rescue cell envelope defects.

### Absence of YhdP and TamB leads to excess LPS shed

OM asymmetry of *yhdP*, *tamB* mutants in W3110 is severely disrupted, yet lowering LPS improves bacterial fitness (**Fig 4D**). The same is true for *E*. *coli* WD101, with decreased LPS levels rescuing WD101 *yhdP* polymyxin resistance and viability of the WD101 *yhdP*, *tamB* mutant. Thus, decreasing LPS restores the OM permeability barrier. Furthermore, both YhdP (1266 aas, 139.1 kDa) and TamB (1259 aas, 136.8, kDa) are very large proteins that are anchored to the IM membrane and could easily span the periplasm. TamB has been predicted to share homology with Vps13, a eukaryotic GPL transporter [5] and during our investigation of YhdP, it was suggested by the Silhavy group to be involved in GPL transport from the IM to the OM [6]. Therefore, if GPL transport to the OM is reduced, lowering LPS synthesis to match the decreased GPL levels in the OM would restore the proper barrier function. Hence, we explored GPL distribution between the membranes in YhdP and TamB mutants.

**Fig 5:**
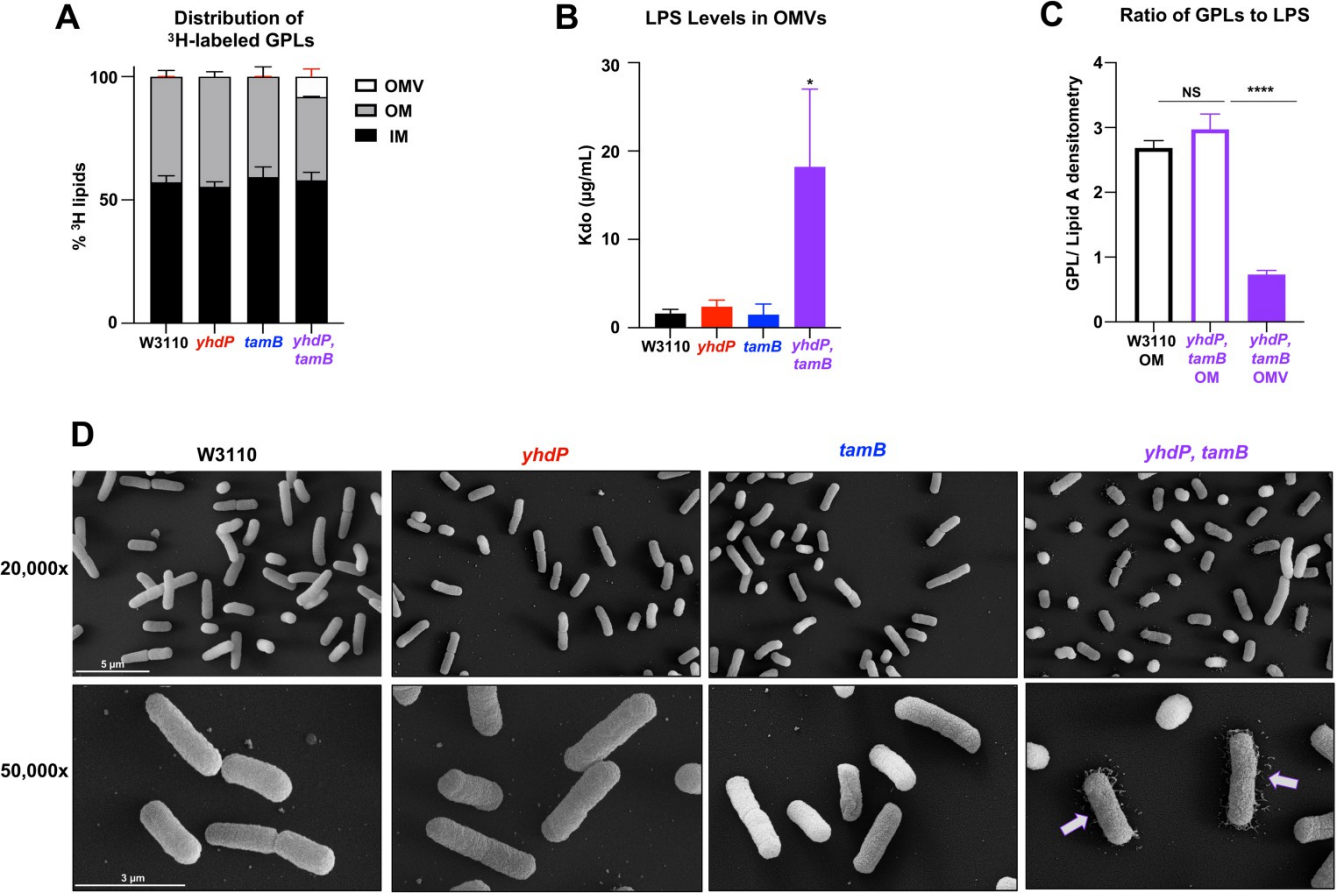
*yhdP*, *tamB* mutants shed OMVs that are rich in LPS. (A) ^3^H-labeled membrane separation. Listed strains were grown to mid-log phase in the presence of ^3^H-glycerol to label GPLs. Inner and outer membrane fractions were separated by a 3-step sucrose gradient and OMVs were collected separately (error bars in red). IM peak fractions are indicated by NADH oxidase and OM peak fractions are indicated by LPS levels (**S2 Fig**). The % of ^3^H-incorporation to indicate GPL levels were performed in biological triplicate. (B) Kdo levels measured from collected OMVs. Growth supernatants were collected, filtered, and OMVs collected by centrifugation. Purpald reagent was used to determine Kdo levels. Error bars represent SD from technical triplicate and is representative of two biological experiments. (C) Total lipid levels of ^32^P_i_ labeled OM and OMV content. WT and *yhdP*, *tamB* cells were grown to mid-log phase and the OM fraction and OMVs were collected and total lipids were extracted for TLC analysis (**S3 Fig**). Error bars represent SD from biological triplicates. 0.001>P***, 0.0001>P****, NS = not significant. (D) Scanning electron microscopy (SEM) of *yhdP*, *tamB* mutants. Top row 20,000x zoom. Bottom row 50,000x zoom. White scale bar set to 5 or 3 μm.

Cells were grown in the presence of [^3^H]-glycerol to label GPLs and the membrane fractions separated by sucrose density gradients. Also, OMVs were collected by ultracentrifugation since this material contains a fraction of the total GPLs once localized to the OM [11, 21]. The IM and OM fractions from *yhdP*, *tamB* and their respective parent strains separated equally across the sucrose gradient as identified by IM (NADH oxidase activity) and OM (LPS) markers (**S2 Fig**) [21]. [^3^H]-GPLs were then extracted by the Bligh-Dyer method from the peak IM and OM fractions and counted by liquid scintillation. There was no major change in GPL distribution between the IM, OM, and OMV material between W3110 and the single *yhdP* or *tamB* mutants (**Fig 5A**). Whereas the *yhdP*, *tamB* mutant showed an ~8% decrease in [^3^H]-counts in the OM compared to wild type. This large decrease in OM GPLs is due to the large increase in shed OMVs that are derived from the OM (**Fig 5A**). We next quantified the amount of LPS in the collected OMVs using the highly sensitive Kdo (3-deoxy-D-*manno*-oct-2-ulsonic acid) assay [3, 22]. WT *E*. *coli* sheds very little LPS, as well as both *yhdP* and *tamB* single mutants (**Fig 5B**). Whereas the *yhdP*, *tamB* mutant sheds ~15x more LPS than wild type, suggesting the OMVs are rich in both GPLs and LPS (**Fig 5B**).

We next investigated the total lipid content of shed OMVs from *yhdP*, *tamB* taking into account the lipid A (LPS) to GPL ratio. Cells were grown in the presence of ^32^P_i_ and OM and OMV fractions were collected. Lipid A and GPLs were isolated in the same extraction, separated by TLC, and subjected to phosphorimaging analysis. Overall, the OM lipid content of WT cells is similar to *yhdP*, *tamB* mutants with a GPL to lipid A ratio of 3:1 (**Figs 5C and S3B and S3C**). However, the GPL:lipid A ratio of OMVs from *yhdP*, *tamB* cells are strikingly different from that of the OM, with a GPL to lipid A ratio of 1:1. Although *yhdP*, *tamB* cells lyse in stationary phase, the OMV fraction was collected in log phase and were not contaminated with IM components as determined by NADH oxidase activity (**S3A Fig**). Thus, the OMV fraction of the double mutant is highly enriched rich with LPS. This was not due to increased LPS synthesis as there were no major changes in LpxC levels in *yhdP*, *tamB* by western blot (**S4A Fig**) [23]. Furthermore, shedding of LPS via OMVs was easily detected by scanning electron microscopy (SEM). The surface of W3110 *yhdP*, *tamB* double mutant has a ruffled appearance compared to wild type and a remarkable level of disassociated cellular material surrounding the bacteria. (**Fig 5D**). Finally, introduction of the *lpxC101* allele into the *yhdP*, *tamB* mutant reduced the amount of shed OMVs (**S4B and S4C Fig**). Overall, this data suggest LPS is lost through OMV shedding to compensate for any decrease in GPL transport to maintain OM lipid homeostasis.

### Absence of YhdP, TamB, and YdbH lead to cell death and GPL accumulation in the inner membrane

**Fig 6:**
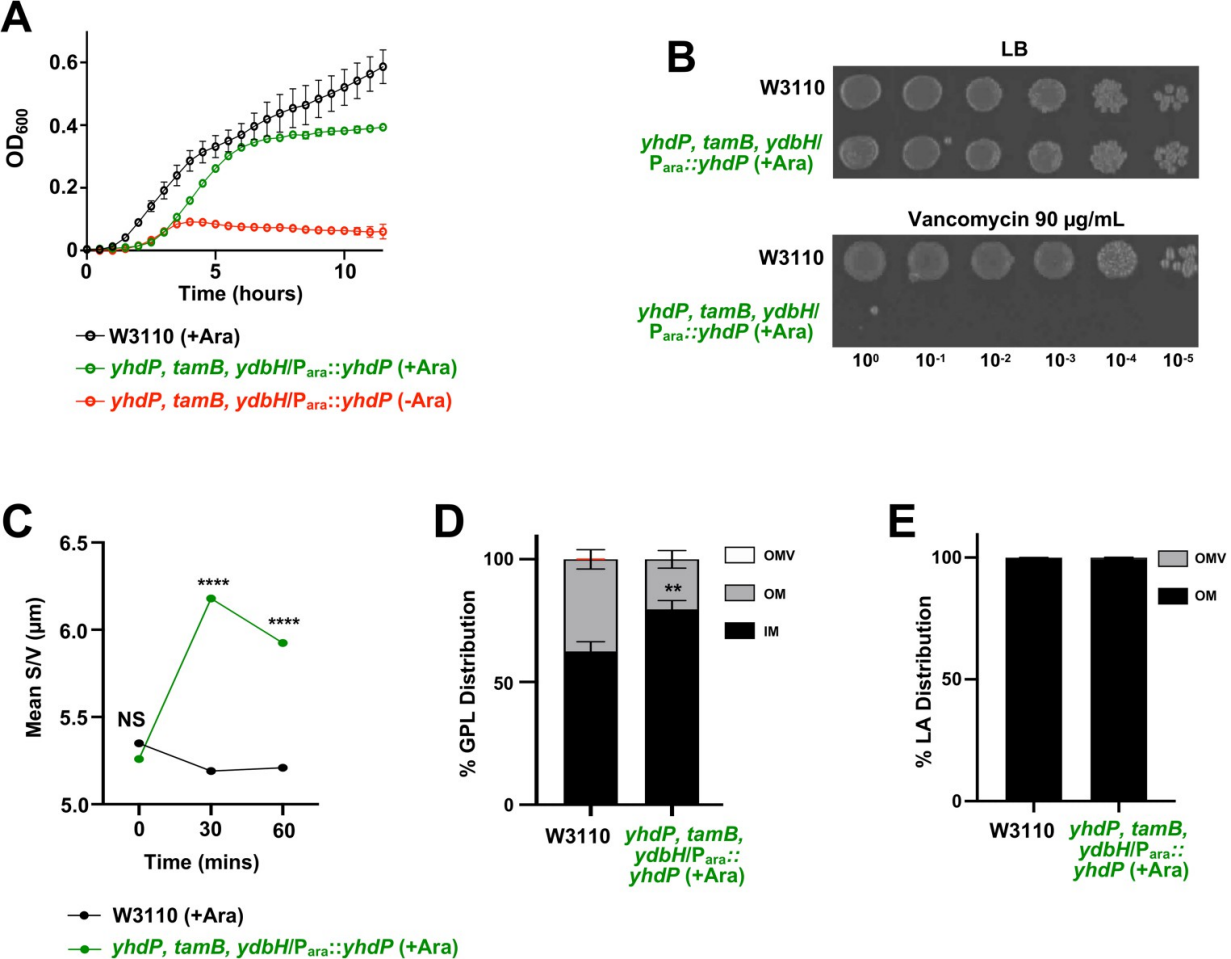
Depletion of AsmA-like functions leads to accumulation of GPLs in the IM. (A) Growth of *yhdP*, *tamB*, *ydbH* mutants. Strains were monitored by OD_600_ every 30 minutes at 37°C and error bars represent SD from technical triplicates. (B) Serial dilutions of indicated strains were spotted on LB plates containing either vancomycin, or no antibiotic. Plates were incubated at 37°C. (C) Surface area/volume measurements of cells at the beginning of ^32^P-labeling after cells grew for 3 hours (**S5B Fig**). Cell width and length of listed strains were measured using MicrobeJ software after strains were inoculated with ^32^P_i_ and imaged at 1000x on agarose pads, S/V was calculated using cell width and length. T-test used between strains. 0.0001>P****. (D) ^32^P-labeled membrane separation. Listed strains were inoculated with ^32^P_i_ after 3 hours of growth for 60 mins. Inner and outer membrane fractions were separated by a 3-step sucrose gradient and OMVs (error bars in red) were collected separately. The % of ^32^P-incorporation indicating GPL levels was performed in biological triplicates. T-test used between strains. 0.01>P**. (E) LPS distribution across OM and OMV. 60 minutes after the start of ^32^P-labeling, lipid A from strains W3110 and W3110 *yhdP*, *tamB*, *ydbH/*P_ara_::*yhdP* grown in arabinose was extracted from the collected OM and OMV fractions and lipid A distribution was calculated from TLC densitometry.

To demonstrate that AsmA-like proteins are involved in GPL transport from the IM to the OM, we attempted to delete the three largest AsmA-like proteins; YhdP (1266 aa), TamB (1259 aa), and YdbH (879 aa) to measure GPL trafficking. We were unable to create a triple mutant suggesting a synthetic lethality. We then transformed W3110 *yhdP*, *tamB* with a plasmid carrying *yhdP* under the control of an arabinose inducible promoter, creating the strain W3110 *yhdP*, *tamB/*P_ara_::*yhdP*. The *ydbH* deletion was then introduced by phage transduction with *yhdP* expressed *in trans*, resulting in the strain W3110 *yhdP*, *tamB*, *ydbH/*P_ara_::*yhdP*. In the presence of glucose, W3110 *yhdP*, *tamB*, *ydbH/*P_ara_::*yhdP* does not grow, but in the presence of arabinose growth is restored, but as predicted not to WT levels (**Fig 6A**).

We first attempted to track GPL transport from the IM to the OM using W3110 *yhdP*, *tamB*, *ydbH/*P_ara_::*yhdP* that was grown in arabinose (inducing conditions) and then shifted to glucose (repressing conditions). Using this method, W3110 *yhdP*, *tamB*, *ydbH/*P_ara_::*yhdP* was able to grow well in both glucose and arabinose for the first 3 hours of growth, at which time we pulsed the strains with ^32^P_i_ to track GPL trafficking (**S5B Fig**). Unfortunately, shortly after the ^32^P-pulse, W3110 *yhdP*, *tamB*, *ydbH/*P_ara_::*yhdP* grown in glucose begins to phenocopy the W3110 *yhdP*, *tamB* strain (**Fig 4B and 4C**) showing significant changes in cell size and loss of excess membrane content from OMVs (after 60 mins of P^32^-labeling) (**S5A and S5C and S5D Fig**). W3110 *yhdP*, *tamB*, *ydbH/*P_ara_::*yhdP* grown in glucose decreases in cell surface area/volume (S/V) overtime. As outlined by Oldewurtel and colleagues [20], cell dimensions of surface area and volume can be utilized to infer changes in dry-mass, as changes in dry-mass are proportional to changes is S/V (**S5C Fig**). Indeed, we observe ~15% of the total lipid A (LPS) lost from the cell surface through OMV shedding. (**S5D Fig**). We therefore found it best to compare W3110 with W3110 *yhdP*, *tamB*, *ydbH/*P_ara_::*yhdP* grown in arabinose for the lipid trafficking assay, because the strain does not shed excess OMVs (**Fig 6D and 6E**). Furthermore, the W3110 *yhdP*, *tamB*, *ydbH/*P_ara_::*yhdP* strain is viable in arabinose yet expression of YhdP alone does not fully complement growth (**Fig 6A**) or support WT levels of vancomycin resistance (**Fig 6B**). This suggests that GPL transport is likely defective when only YhdP serves as the primary transport system.

At the initial ^32^P_i_ pulse of the W3110 and W3110 *yhdP*, *tamB*, *ydbH/*P_ara_::*yhdP* cells grown in arabinose, the S/V is not significantly different, suggesting the cells have the same dry-mass (**Fig 6C**) [20]. But after 30 and 60 minutes following the addition of ^32^P_i_, the S/V of the bacteria increases, while the W3110 S/V remains stagnant, suggesting the dry-mass of W3110 *yhdP*, *tamB*, *ydbH/*P_ara_::*yhdP* cells is increasing (**Fig 6C**). This increase in S/V for the mutant is coupled with a striking increase in accumulation of GPLs in the IM (**Fig 6D**) with an ~20% increase of IM GPLs when only YhdP is present. Importantly, there was no change in lipid A (LPS) distribution with essentially all of the ^32^P-labeled lipid A found in the OM fraction and not in released OMVs. Overall, these data suggest that absence of AsmA-like proteins directly impact anterograde transport of GPLs to the OM.

## Discussion

**Fig 7:**
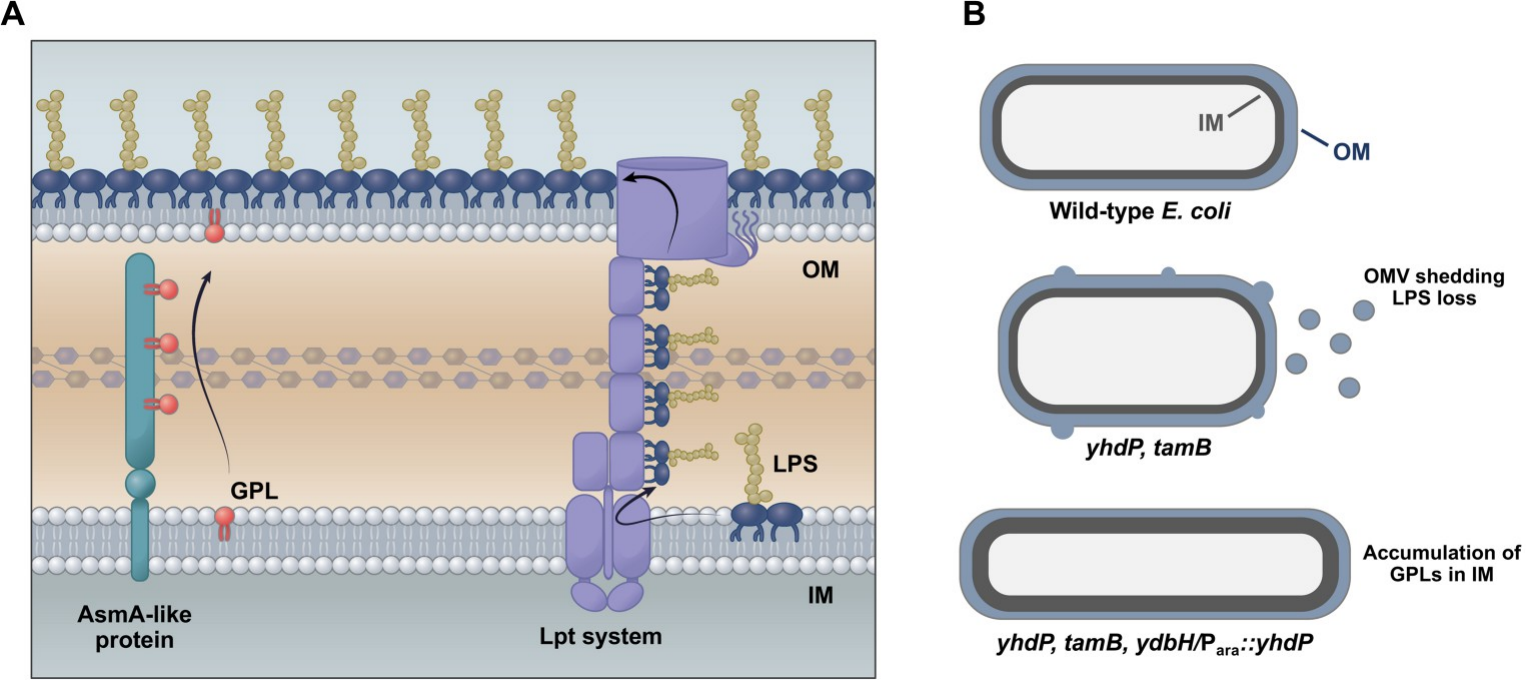
Model of lipid transport to the OM. (A) The Lpt system transports LPS from the IM to the outer leaflet of the OM. Data presented here suggests that GPL transport to the inner leaflet of the OM from the IM is accomplished by the dominant AsmA-like proteins; YhdP, TamB, and YdbH. (B) Schematic of general envelope defects in AsmA-like mutants. Top: WT *E*. *coli* with IM in grey and OM in blue. Middle: In the absence of YhdP and TamB, excess LPS is shed from the OM to maintain appropriate balance between GPLs and LPS in the OM along with a change in cell shape to accommodate reduced membrane content. Bottom: When only the dominant GPL transporter (YhdP) is expressed, transport is maintained at levels that no longer requires LPS shedding for cell viability. However, GPL transport is slowed leading to accumulation of GPLs in the IM and changes in cell shape support excess membrane content.

The unique organization and adaptability of the OM allows Gram-negative bacteria to resist large polar molecules and amphipathic compounds, such as the polymyxin antibiotics [1]. Multiple complexes work synergistically to assemble and maintain the unique OM structure and asymmetry. The Lol complex shuttles lipoproteins destined to the OM, whereas the Lpt system exports LPS to the OM [24]. Also, a variety of systems exist to remove or degrade mislocalized GPLs from the outer leaflet of the OM. PagP acylates lipid A using a palmitate from a GPL donor as a substrate, increasing acyl chain packing, whereas PldA acts as a lipase degrading GPLs [24]. One system for removal of mislocalized GPLs that has been studied extensively is the Mla system. The Mla system functions to transport GPLs from the outer leaflet of the OM to the IM [24, 25]. The directionality of Mla mediated GPL transport has been the topic of debate, but recent publications have expanded our understanding of the Mla system, implicating that Mla’s function is to mediate retrograde GPL transport [26–28]. Still, how GPLs are transported to the OM from the IM has remained elusive. Using polymyxin to probe for genes required for high-level antibiotic resistance, we demonstrate here that members of the AsmA-like family, YhdP, TamB, and YdbH are required for maintaining GPL levels in the OM, suggesting a role of GPL transport (**Fig 7**).

To identify novel genes essential for polymyxin resistance and OM biogenesis, we assessed the fitness of a highly saturated Tn-library from a polymyxin-resistant *E*. *coli* strain (WD101) in the presence of a sublethal dose of polymyxin. Multiple genes were identified by Tn-seq. (**Fig 1B**). Polymyxin’s cationic charge allows the drug to bind to the cell surface via the negatively charged lipid A leading to eventual disruption of the cell envelope [29]. Consequently, bacteria modify their LPS to mask the negative charges of lipid A, resulting in bacteria to become polymyxin resistant [29]. Interestingly, loss of YhdP does not alter lipid A modifications (**Fig 1D**), but instead results in general OM defects as evidenced by increased sensitivity to vancomycin and agents that perturb OM structure (e.g. SDS-EDTA) [7, 8]

In this study we bolster the claim that YhdP [6], and other AsmA-like proteins, are involved in GPL transport. We begin by demonstrating polymyxin resistance is restored in WD101 *yhdP* when LPS levels are lowered (**Fig 2B and 2C**). We hypothesize that decreasing LPS synthesis allows the cell to maintain OM asymmetry by matching the decrease in GPL transport to the OM. Since *yhdP* is not essential for viability (**Fig 2A**), we speculated that GPL transport might be redundant in *E*. *coli*. Therefore, we expanded our studies into the AsmA-like family and found that a double *yhdP*, *tamB* mutant is lethal in WD101 (**Fig 3B**). Here again, lowering LPS levels rescues the observed phenotype and restored growth of the synthetically lethal *yhdP*, *tamB* WD101 strain (**Fig 3C**).

YhdP is the largest protein (1266 amino acids) of the AsmA-like family, whereas TamB is the second largest (1259 amino acids). TamB has been identified to share homology with the eukaryotic GPL transporter Vps13, and contains a large hydrophobic interior that could accommodate GPLs [5, 30]. To study any possible additive effects, we moved to a W3110 background and observed that loss of both proteins resulted in cell lysis in stationary phase, changes in cell morphology, severe vancomycin sensitivity and excess OMV shedding (**Figs 4 and S4B and S4C**). These phenotypes could be partially restored by lowering LPS levels. Presumably, cell lysis occurs due to the reduction in GPL transport without a concomitant decrease in LPS synthesis (**S4A Fig**)*—*conditions that would perturb OM homeostasis and result in antibiotic sensitivity. Interestingly, the cell seems unable to “sense” a change in the level of OM GPLs as the *yhdP*, *tamB* double mutant does not respond by decreasing LPS synthesis. Instead, to maintain OM integrity, we found that excess LPS is lost in OMVs (**Fig 5C and 5D**). Following fractionation of cell envelope components, we determined that *yhdP*, *tamB* mutants have a GPL:LPS ratio of 3:1 in the OM fraction, like wild type, whereas the OMV fraction has a GPL:LPS ratio of 1:1 (**S3A Fig**). Thus, LPS is transported at a higher rate to the OM compared to GPLs when key AsmA family members are absent, even though LPS synthesis does not increase. Since all GPLs shed in OMVs have undergone anterograde transport from the IM [27], this data supports that *yhdP*, *tamB* mutants are defective in GPL transport.

Deletion of *ydbH* that encodes the third largest AsmA-like protein (YdbH, 879 aa) in *yhdP*, *tamB* mutants is synthetically lethal (**Fig 6A**). Plasmid-based expression of YhdP in the triple mutant does not rescue vancomycin resistance or completely restore growth (**Fig 6A and 6B**); however, these cells no longer shed excess LPS through OMVs (**Fig 6E)** allowing us to measure GPL transport without heightened OMV production. Under these conditions, we observed an accumulation (~20%) of GPLs in the IM compared to the WT control (**Fig 6C and 6D**), supporting a role for AsmA-like proteins in lipid transport. When only YhdP is available for lipid transport, we observed visibly longer cells (**S5A Fig**). This is consistent with cells that accumulate OM lipids at the IM [11, 31, 32].

AsmA-like proteins are found in all phyla containing an OM, with the exception of *Thermotogae* and *Thermodesulfobacteria* [33]. *E*. *coli* contains a total of 6 AsmA-like proteins, but our data suggest that only the three largest proteins (AsmA, TamB, YdbH) share a redundant function in GPL transport. Interestingly, both TamB and YdbH are in an operon with an OM protein, whereas YhdP lacks any obvious associated OM partner. TamB interacts with the OM protein TamA, a homologue of BamA that is the major component of the Bam complex required for assembly of OM β-barrel proteins. The TamAB complex was previously associated with the assembly of a subset of OM proteins, including key autotransporters [34]. However, TamB is far more widely distributed then TamA [33] supporting a role for TamB beyond OM protein assembly. The previous observation that TamB plays a role in OM protein assembly should be investigated further given that TamB plays an important role in OM lipid homeostasis.

A portion of the C-terminus domain of TamB has been crystalized and reveals a structure that is rich with β-strands forming a hydrophobic interior referred to as the “β-taco fold”, a feature that is striking similar to β-jellyroll architecture found in proteins of the Lpt system [30]. Whereas the N-terminus of TamB and AsmA have homology with the Chorein-N domain of VSP13, a eukaryotic protein that contains a hydrophobic lining used for GPL transport [5]. VSP13 bridges eukaryotic organelles to mediate lipid transfer, this protein is anchored to the membrane by a N-terminal α-helix which is followed by a conserved Chorein-N domain. Lipids are hypothesized to shuttle through the remaining protein structure that is rich in β-strands [35, 36]. The overall architecture of the GPL transporter VSP13 is strikingly similar to YhdP, TamB, and YdbH. Lastly, YhdP has been implicated in GPL transport as loss of YhdP slows IM rupture in a high flux GPL transport background [6]. Furthermore, a recent publication also reveals the essentiality and redundancy of YhdP, TamB, and YdbH [37]. The authors also demonstrate a disruption in OM lipid homeostasis in *yhdP tamB* mutants, but do not establish defects in GPL transport [37]. Given the work presented here and the impressive work of our colleagues [6, 37], it is clear that this remarkable family of proteins plays an important role in cell envelope biogenesis. Our data reveals the accumulation of GPLs in the IM and excess LPS shuttled to the OM in the absence of these proteins, providing a model where the AsmA clan is responsible for the highly sought-after anterograde GPL transport system.

## Material and methods

### Bacterial growth conditions

Bacteria cultures were grown in LB or LB agar at 37°C. LB was supplemented with ampicillin (Amp) (100 μg/mL), kanamycin (Kan) (30 μg/mL), L-arabinose (0.2% [w/v]), and/or D-glucose (0.2% glucose [w/v]). For growth curves, the BioTek Epoch2 plate reader was used to grow and monitor 0.2 mL cultures in a polystyrene 96-well plate.

### Strain construction

All bacterial strains, plasmids, and oligonucleotide primers used in this study are listed in S1 Table. Mutations were introduced into the chromosome of *E*. *coli* K-12 strain W3110 using generalized transduction and the Keio collection [38]. Flippase/flippase recognition target (FLP/FRT) site-specific recombination was used to remove FRT-flanked resistance cassettes [39]. pCP20, which expresses FLP from a temperature-sensitive promoter, was electroporated into W3110 containing the Keio allele. Transformants were recovered in LB at 30°C for 1 h followed by selection on LB agar supplemented with Amp at 30°C. The following day, single colonies were grown on LB at 37°C. Colonies were screened on Amp and Kan for sensitivity to confirm loss of both pCP20 and removal of the *kan* allele. PCR was used to confirm each mutation and removal of the Kan resistance cassette. The scar region following FLP/FRT recombination contains a single FRT site [40].

### Generation of the *E*. *coli* WD101 Tn mutant library

The construction of the Tn mutant library in WD101 was done as previously described [11]. Briefly, a donor strain was created in *E*. *coli* β3914, a diaminopimelic acid (DAP) auxotroph, by electroporation with pJNW684 [10]. Tn libraries were then generated by mating WD101 with β3914, exconjugants were selected on Kan. A total of 450,000 exconjugant colonies were collected by scrapping agar plates and the colonies pooled and stored in 30% glycerol at -80°C.

### Growth challenge assay and DNA library preparation

Three individual aliquots of the Tn library were thawed and back-diluted into 50 mL of plain LB with or without 5 μg/mL of polymyxin B at a starting OD_600_ (optical density at 600 nm) of 0.001 and grown at 37°C until an OD_600nm_ of 0.5. Cells were collected and genomic DNA was extracted with the use of the Easy-DNA kit from Invitrogen following manufacturer instructions. DNA was then concentrated at 250 ng/μl and sheared by sonication to obtain fragments around 300 bp. 2.5 μg of sheared DNA was then used for Poly-C tail addition using a terminal deoxynucleotidyl transferase (Promega) for 1 h at 37°C using 9.5 mM dCTP/0.5 mM ddCTP mix per manufacturer instructions. DNA fragments were purified using AMPure beads (Beckman Coulter) and used as template for a first PCR step using Platinum Pfx polymerase (Invitrogen) along with primers olj510-Biotin and olj376. PCR products were again purified using AMPure beads, and the biotin-tagged eluted DNA was separated using streptavidin beads (New England Biolabs). Before the DNA purification, the streptavidin beads were equilibrated in 1X binding/wash buffer (1 M NaCl, 5 mM Tris-HCl, 0.5 mM EDTA, pH 7.5), and washed with 1X binding/wash buffer and two washes in LOTE buffer (Low salt Tris EDTA, 3 mM Tris-HCl 0.2 mM EDTA, pH 7.5). The biotin-tagged DNA bound to the streptavidin beads was used as template for a second PCR step, using the Platinum Pfx Polymerase and primers olj511 and BC#. The PCR product was purified using 40 μl of AMPure beads, and the concentration of the DNA quantified using the Qubit dsDNA High Sensitivity assay and the Qubit 3.0 Fluorometer (Life technologies). Samples were paired-end sequenced using Illumina HiSeq 2500 platform at the Georgia Genomics and Bioinformatics Core Facility. Tn-Seq data analysis was performed using QIAGEN CLC Genomics Workbench and the *E*. *coli* W3110 genome sequence (GenBank accession number NC_007779).

### Efficiency of plating

Overnight bacterial cultures were standardized by OD_600_ and serially diluted by a factor of 10 in a 96-well plate in LB. Bacteria were then transferred using a 96-well plate replicator onto LB plates with the indicated antibiotics.

### Determination of polymyxin minimum inhibitory concentration (MIC)

MICs were determined by E-strip (BioMerieux). Cultures grown to mid-log phase were back diluted 10-fold and spread on LB plates. Once the plate dried, sterile E-strips were added and incubated overnight at 37°C. The MIC was assigned as the value where the zone of inhibition intersected with the E-strip.

### Analysis of ^32^P-labeled lipid A

Isolation of ^32^P-labeled lipid A was carried out as previously described [41, 42]. Briefly cultures were grown in 2.5 μCi/mL of ^32^P ortho-Phosphoric acid (^32^P_i_) (Perkin-Elmer) to an OD_600_ of 0.8 to 1.0. Lipid A was extracted via mild-acid hydrolysis followed by Bligh-Dyer solvent extraction as previously described [11]. TLC migration of lipid A samples was performed in a pyridine, chloroform, 88% formic acid, aqueous (50:50:16:5 v/v) solvent system. Plates were exposed to a phosphorscreen and imaged.

### SDS-PAGE and LpxC immunoblotting

The IM protein, LpxC, was used to quantitate LPS synthesis. Boiled fractions in SDS were loaded in equal volumes (10 μL) and analyzed by SDS-PAGE using a 10% Bis-Tris gel (Fisher). Western blot analysis was carried out via a gel transfer to a low fluorescent polyvinylidene fluoride (PVDF) membrane (Thermo Scientific) using the Novex Xcell II Blot Module. All blots were blocked overnight in a 2% ECL prime blocking agent. The blot was probed with two antibodies, one probing for LpxC and the second for detection of the glyceraldehyde 3-phosphate dehydrogenase (GapDH) that served as an internal loading control. The primary rabbit polyclonal α-LpxC [a generous gift from the Doerrler Laboratory, Louisiana State University [43], 1:10,000 dilution] was used in conjunction with a polyclonal goat anti-rabbit cyanine5 as the secondary and the mouse monoclonal α-GapDH (Fisher Scientific, 1:10,000 dilution) used with a polyclonal goat anti-mouse cyanine3. Blots were imaged on a Typhoon NIR Plus (Amersham).

### LPS quantification

Strains of interest were grown to mid-log phase, standardized by OD_600_ and harvested in a microcentrifuge. Boiled fractions in LDS were treated with proteinase K to remove contaminating proteins as previously described [11]. Samples were loaded in equal volumes (10 μL) and analyzed by SDS-PAGE using a 10% Bis-Tris gel (Fisher). Pro-Q Emerald 300 stain kit was used following the manufactures instructions to visualize the LPS. Images were captured on a BIORAD ChemiDoc MP Imaging System. To avoid image saturation and ensure samples were analyzed in the linear range of detection, proteinase-K treated lysate from the W3110 control was used to perform a standard curve. Standardization of samples using total protein in cell lysates (prior to proteinase K treatment), instead of OD_600_, provided similar results.

### ^32^P-labeling of conditional triple mutant

W3110 pBAD and W3110 *yhdP*, *tamB*, *ydbH/*P_ara_::*yhdP* were grown over night at 37°C in LB broth containing 0.2% arabinose. Overnight cultures were back diluted 1:100 in LB broth containing 0.2% arabinose, once OD_600_ of 1.0 was reached, cultures were once again back diluted to a starting OD_600_ of 0.02 in LB broth supplemented with either 0.2% arabinose or glucose. After 3 hours of growth, 2.5 μCi/mL [^32^P_i_] was added to the culture. 10 mL aliquots were collected and mixed with 50 μM of CCCP to halt GPL transport. Membrane separations and OMVs were then processed next.

### Membrane separations

Sucrose density gradient centrifugation were used to separate IM and OM fractions as previously described [11, 21]. For each strain, 30 mL cultures were grown to mid-log phase labeled with either 2 μCi/mL of [^3^H]-glycerol (Perkin-Elmer) or 2.5 μCi/mL of ^32^P_i_ (Perkin-Elmer). Cells were harvested at 5,000 x g for 10 minutes and where appropriate, the supernatant saved for collection of shed OM material. The resulting cell pellet was suspended in 10 mM Bis-Tris pH 8.0 and centrifuged again. Cells were then resuspended in 6 mL of 10 mM Bis-Tris pH 8.0, 20% sucrose (w/w) and lysed by single passage through a mechanical cell press at 8,000 psi. Unbroken cells were removed by centrifugation at 5,000 x g for 10 minutes. Cell lysate was then layered on top of a two-step gradient consisting of 1.5 mL of 65% sucrose (w/w) and 5 mL of 40% sucrose (w/w). Samples were centrifuged at ~100,000 x g 16 hours, 0.8 mL fractions were then collected from the top.

### NADH oxidase assay

The inner membrane (IM) enzyme, NADH oxidase, was used as a marker for the IM as previously described [21]. Briefly, 2.5 μL of each fraction from the sucrose density gradient was added to a 96-well black bottom plate containing 180 μL of 100 mM Tris HCl, pH 8.0 containing 120 μM NADH (Sigma) and 5 mM dithiothreitol (DTT, Sigma) per well. Changes in fluorescence over time with an excitation at 340 nm and emission at 465 nm was monitored. The activity of NADH oxidase for each fraction is represented as a % of total NADH oxidase activity across the gradient.

### Quantification of OMVs

Cultures were pelleted at 5,000 x g for 10 minutes and the supernatant collected. This spin was repeated with the supernatant to remove cellular debris. The twice-spun supernatant was passed through a 0.22 μm filter and stored at 4°C. Supernatant was then centrifuged at 100,000 x g at 4°C for a minimum of 3 hours. The washed pellet containing shed OM material was resuspended in phosphate-buffered saline. Shed OM was quantified using the colorimetric Purplad assay, as previously described [22]. Values were measured using an H1 Hybrid Plate Reader (Biotek) and converted to μg/mL of Kdo using a standard curve generated with pure Kdo.

### Total lipid analysis

Lipid A and GPLs extracted from sucrose gradient membrane fractions were collected as previously described [31]. Briefly, 200 μL of pooled peak membrane fractions were diluted in 1.6 mL of 12.5 mM sodium acetate, pH 4.5 with 1% SDS and boiled for 30 minutes. The hydrolysis reaction releases the lipid A anchor from any LPS present in the membrane gradient fraction, while not impacting GPL species. Bligh-Dyer solvent extraction was then used to collect total lipids, both freed lipid A and GPLs. TLC migration was then conducted as described above to determine the GPL to lipid A ratio.

### Phase contrast microscopy

Cultures grown to mid-log phase in LB and spotted on 3% agarose pads. 10 fields of view were captured with the Olympus CX43 equipped with an Olympus Infinity 355 camera in phase contrast. Cell size was determined with the MicrobeJ [44]. Surface area was calculated using the following equation: S = 4π(W/2)^2^ + 2πl. Surface area/volume was calculated using the following equation: S/V = 4/[W(1 –W/3L)]. Where W represents width and L represents length.

### Scanning electron microscopy

Cultures grown to late-log phase in 5 mL LB were pelleted at 5,000 x g for 10 minutes and the supernatant was discarded. Sample pellets were fixed in 2% glutaraldehyde 0.1M cacodylate buffer for 2 hours at room temperature. After fixation, samples were washed in 0.1M cacodylate buffer. Samples were then post-fixed in 1% OsO_4_ 0.1M cacodylate buffer for 1 hour at room temperature and then washed with water. Samples were then dehydrated with an ethanol series for 10 minutes at each step (25, 50, 75, 100% ethanol). Once samples were suspended in 100% ethanol, they were placed on poly-L-lysine coated glass cover slips. Samples were then critical point dried, mounted on aluminum SEM stubs and sputter-coated with gold-palladium. Images were taken on a Teneo FE-SEM.

## Supporting information

S1 Fig Antibiotic resistance of *yhdP* suppressors. Panels A and B. Serial dilutions of indicated strains were spotted on LB plates containing either polymyxin, vancomycin, or no antibiotic. Plates were incubated at 37°C.
(TIF)

S2 Fig Membrane marker analysis of IM and OM separations. Every fraction from each strain listed was analyzed for the level of [^3^H]-glycerol incorporation (GPLs), NADH oxidase activity, and the presence of LPS. The NADH oxidase activity and presence of LPS were used to validate efficient separation of IM and OM fractions.
(TIF)

S3 Fig Analysis of the total lipid content (GPLs and lipid A) of the OM and OMV fractions of *yhdP*, *tamB*. (A) OMV preparations isolated from *yhdP*, *tamB* mutants were subjected to NADH oxidase assay to determine any possible IM contamination that could arise from cell lysis. Whole membrane preparations, containing both IM and OM, served as a positive control showing robust NADH oxidase activity. Error bars represent SD from technical triplicates that are representative of 2 biological experiments. (B) W3110 and the *yhdP*, *tamB* mutant were grown to mid-log and the OM and OMV fractions were collected and a total lipid extraction (lipid A + GPLs) performed. Lipids were separated and analyzed by TLC. (C) Densitometry of TLC shown in panel B indicating the level of lipid A and GPL species. Data in panels B and C are representative of biological triplicates.
(TIF)

S4 Fig LpxC expression levels and the effect of lowering LPS levels in *yhdP*, *tamB*. (A) LpxC levels were analyzed by SDS-PAGE and western blot. LpxC density levels were measured and normalized to GapDH loading control. The final level of LpxC shown is compared that of WT (W3110). Error bars represent SD from three biological experiments (B) Kdo levels measured from collected OMVs. Supernatant was collected, filtered, and OMVs were pelleted. Purpald reagent was used to determine Kdo levels. Error bars represent SD from technical triplicate and is representative of two biological experiments. (C) Surface area/volume measurements of cells at late log growth. Cell width and length of listed strains were measured using MicrobeJ software and imaged at 1000x on agarose pads, S/V was calculated using cell width and length. T-test used between strains. 0.05>P*, 0.0001>P**** NS = Not Significant.
(TIF)

S5 Fig Depletion of YhdP in a *yhdP*, *tamB*, *ydbH* triple mutant results in release of excess LPS and changes in cell shape. (A) Microscopy of *yhdP*, *tamB*, *ydbH* triple mutant expressing YhdP from an arabinose-inducible promoter (W3110 *yhdP*, *tamB*, *ydbH/*P_ara_::*yhdP)*. Phase contrast microscopy (1000x) of the indicated cells with white scale bar set to 5 μm. Image in bottom left corner of dividing cell is a 300x zoom of the larger image. Microscopy data is representative of two biological experiments. (B) Growth of *yhdP*, *tamB*, *ydbH* mutants. Strains were monitored by OD_600_ every hour at 37°C. After 3 hours of growth, cultures were inoculated with ^32^P_i_ and aliqouts were collected for further analysis. Error bars represent SD from biological triplicates. (C) Surface area/volume measurements of cells at beginning of ^32^P_i_ addition. Cell width and length of listed strains were measured using MicrobeJ software after strains were inoculated with ^32^P_i_ and imaged at 1000x on agarose pads, S/V was calculated using cell width and length. T-test used between strains. 0.0001>P****. (D) LPS distribution across OM and OMV. 60 minutes after W3110 and W3110 *yhdP*, *tamB*, *ydbH/*P_ara_::*yhdP* grown in glucose was inoculated with ^32^P_i_, lipid A was extracted from the collected OM and OMV fractions and lipid A distribution was calculated from TLC densitometry. T-test used between strains. 0.05>P*.
(TIF)

S1 Table Strains, plasmids and primers used in this study. (DOCX)
