# Supplementary figures and images for: Absence of YhdP, TamB, and YdbH leads to defects in glycerophospholipid transport and cell morphology in Gram-negative bacteria
Source: PLoS Genet. 2022 Feb 28;18(2):e1010096. doi: 10.1371/journal.pgen.1010096 (PMC8912898; doi:10.1371/journal.pgen.1010096)

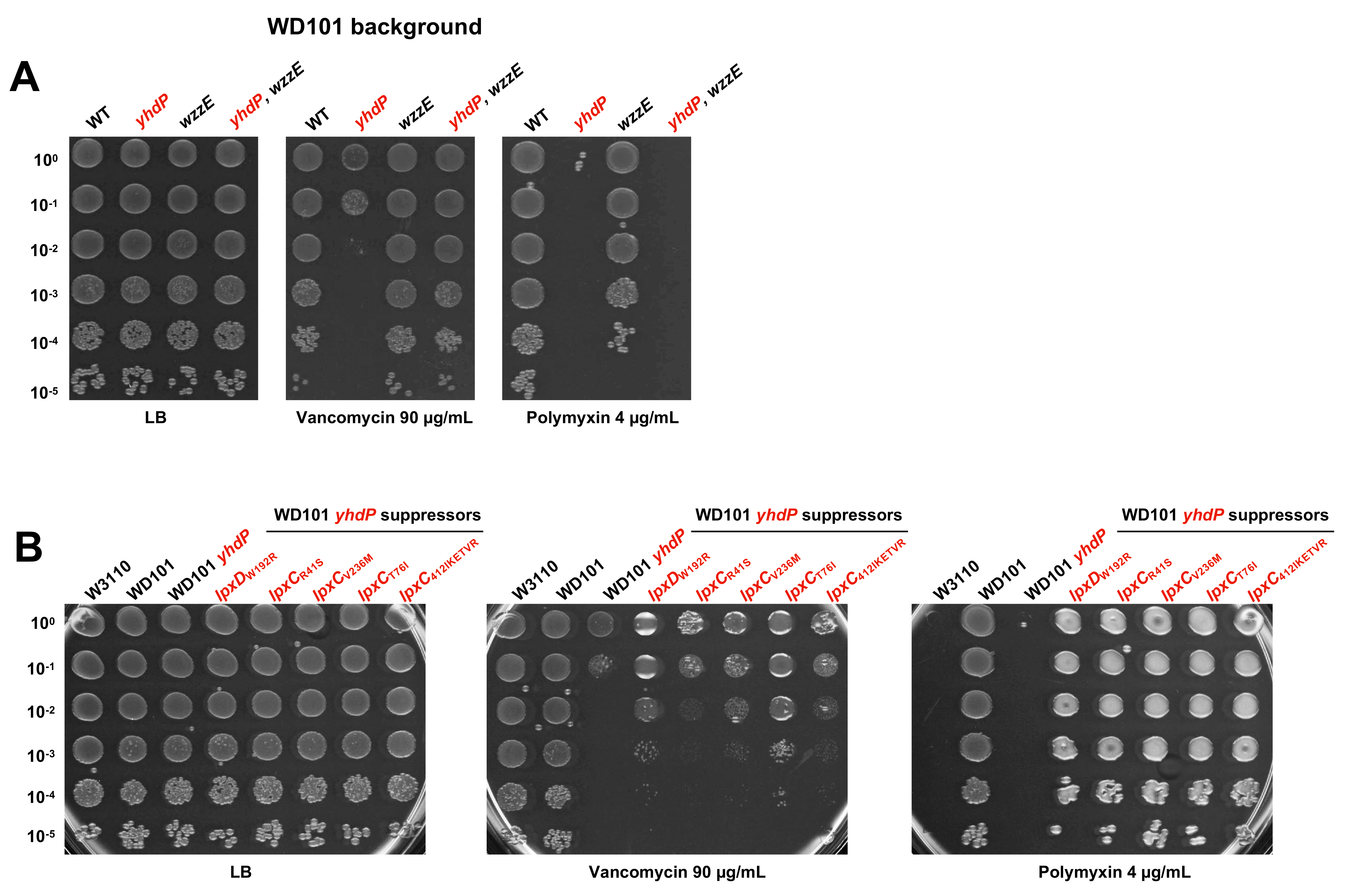

Supplement: S1 Fig — Panels A and B. Serial dilutions of indicated strains were spotted on LB plates containing either polymyxin, vancomycin, or no antibiotic. Plates were incubated at 37°C. (TIF) [file pgen.1010096.s001.tif]

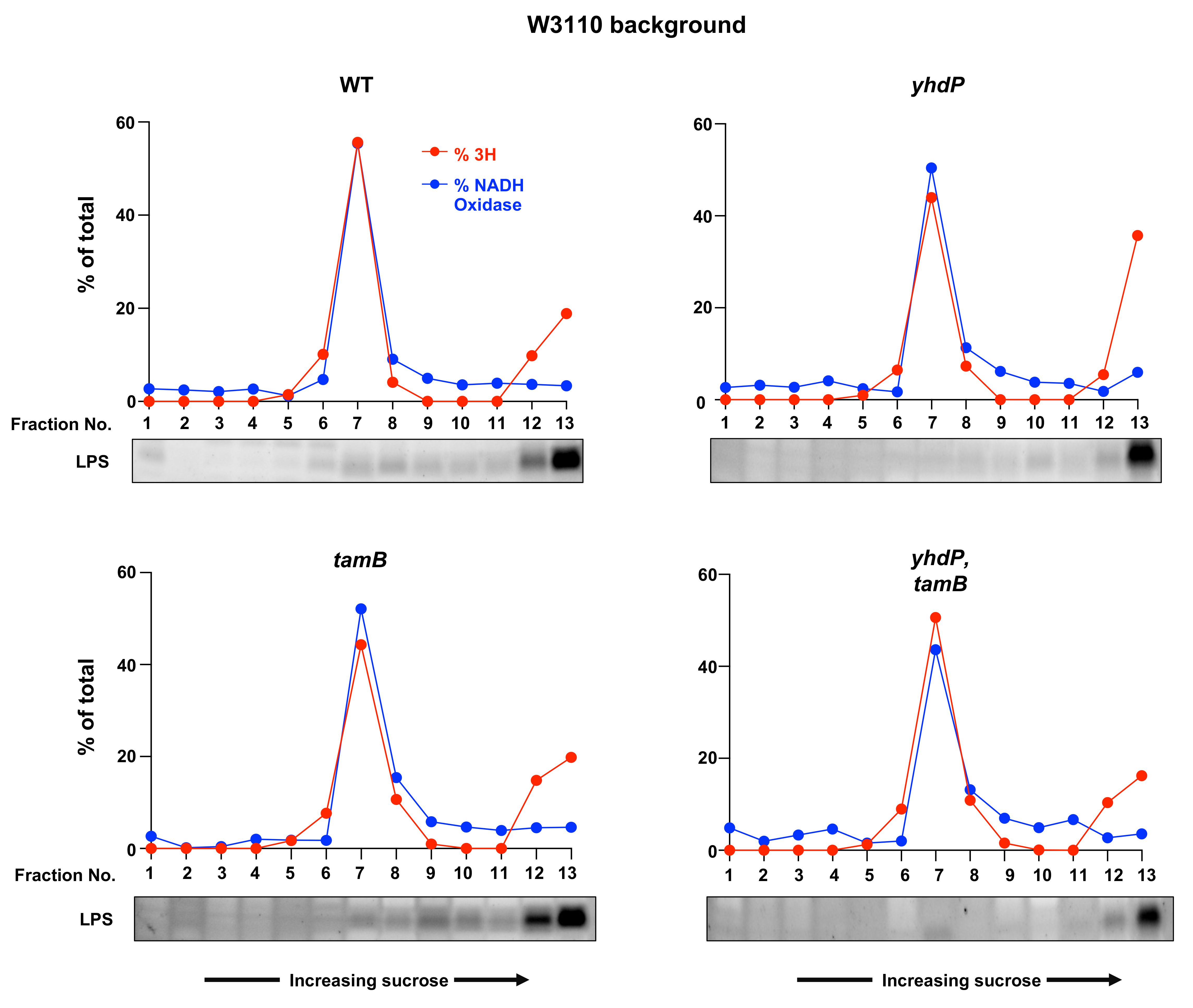

Supplement: S2 Fig — Every fraction from each strain listed was analyzed for the level of [3H]-glycerol incorporation (GPLs), NADH oxidase activity, and the presence of LPS. The NADH oxidase activity and presence of LPS were used to validate efficient separation of IM and OM fractions. (TIF) [file pgen.1010096.s002.tif]

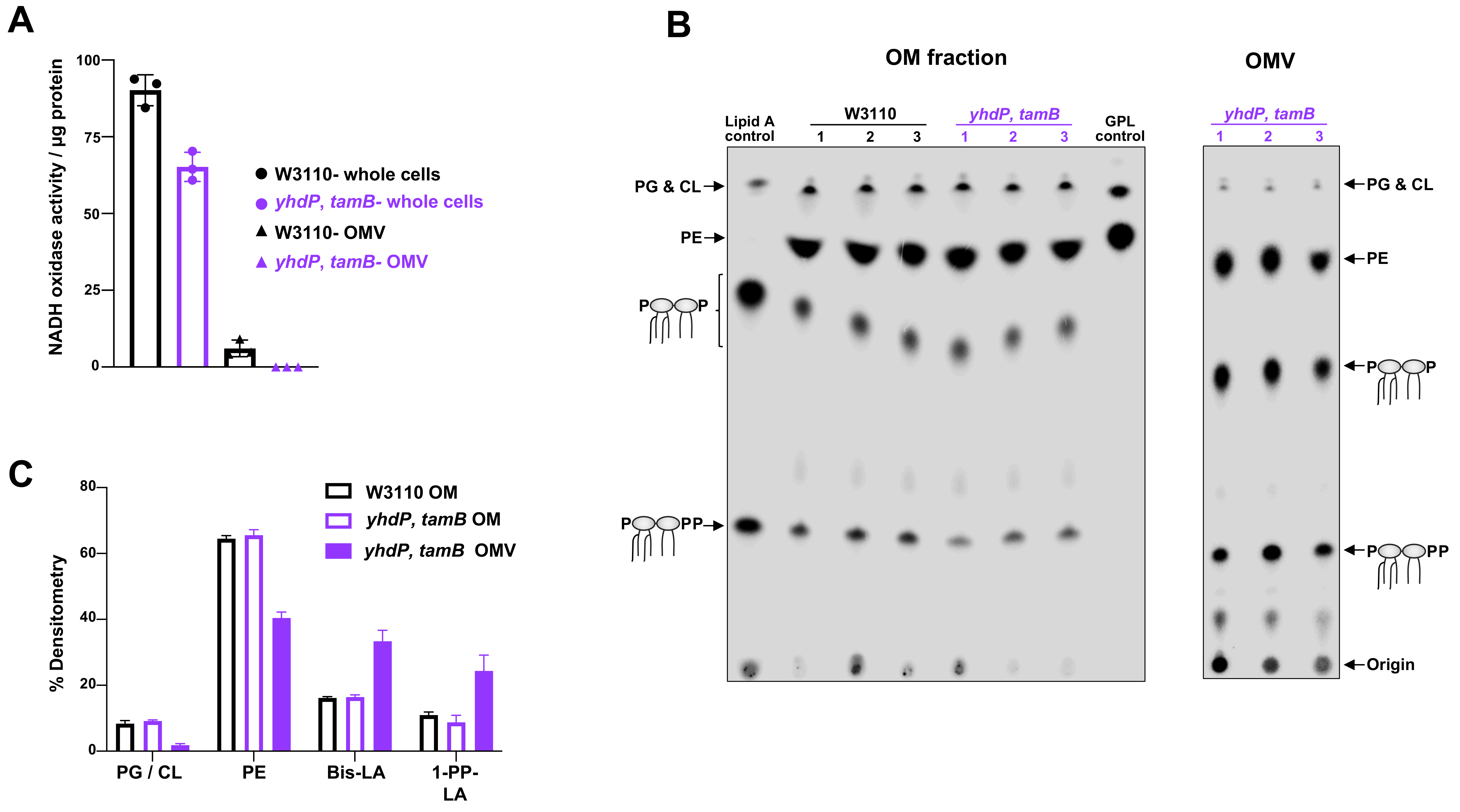

Supplement: S3 Fig — (A) OMV preparations isolated from yhdP, tamB mutants were subjected to NADH oxidase assay to determine any possible IM contamination that could arise from cell lysis. Whole membrane preparations, containing both IM and OM, served as a positive control showing robust NADH oxidase activity. Error bars represent SD from technical triplicates that are representative of 2 biological experiments. (B) W3110 and the yhdP, tamB mutant were grown to mid-log and the OM and OMV fractions were collected and a total lipid extraction (lipid A + GPLs) performed. Lipids were separated and analyzed by TLC. (C) Densitometry of TLC shown in panel B indicating the level of lipid A and GPL species. Data in panels B and C are representative of biological triplicates. (TIF) [file pgen.1010096.s003.tif]

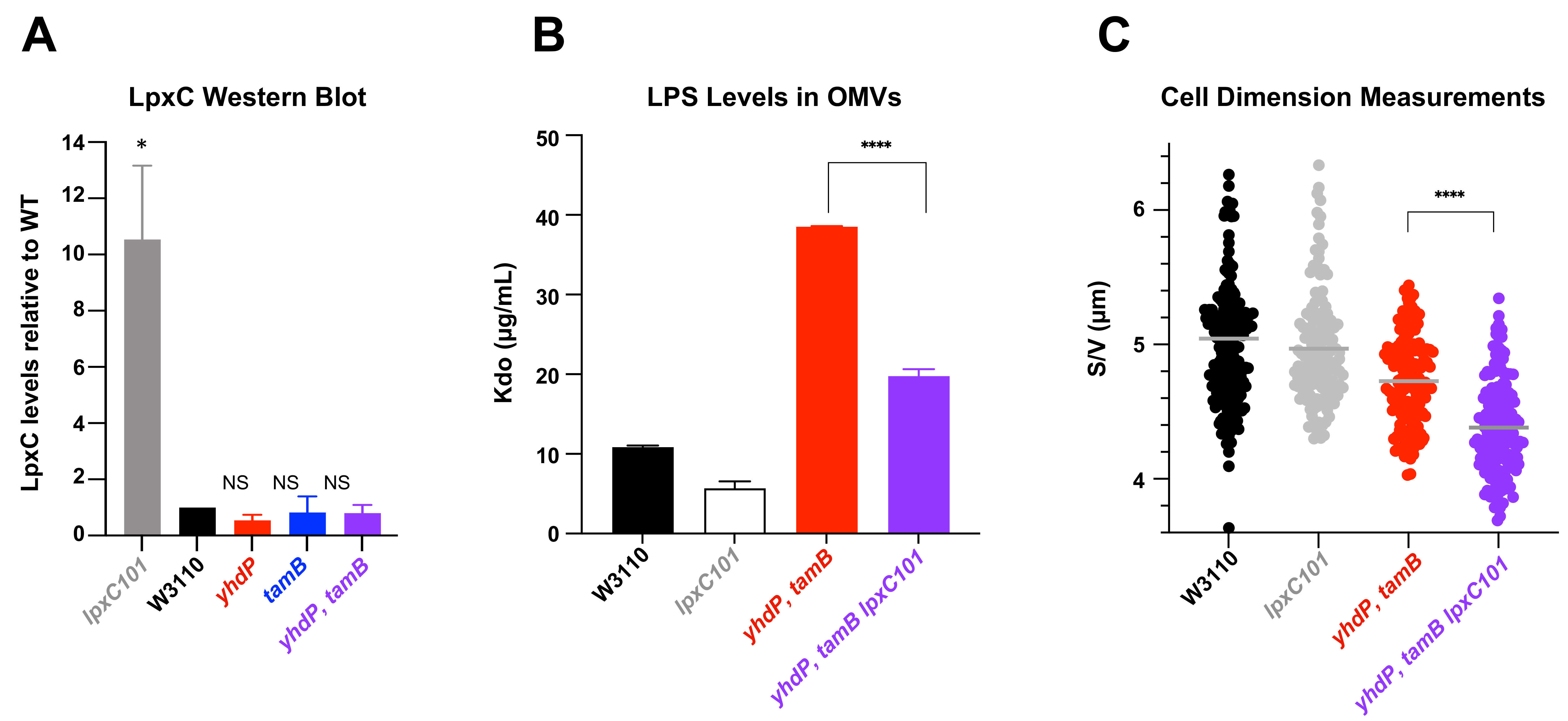

Supplement: S4 Fig — (A) LpxC levels were analyzed by SDS-PAGE and western blot. LpxC density levels were measured and normalized to GapDH loading control. The final level of LpxC shown is compared that of WT (W3110). Error bars represent SD from three biological experiments (B) Kdo levels measured from collected OMVs. Supernatant was collected, filtered, and OMVs were pelleted. Purpald reagent was used to determine Kdo levels. Error bars represent SD from technical triplicate and is representative of two biological experiments. (C) Surface area/volume measurements of cells at late log growth. Cell width and length of listed strains were measured using MicrobeJ software and imaged at 1000x on agarose pads, S/V was calculated using cell width and length. T-test used between strains. 0.05>P*, 0.0001>P**** NS = Not Significant. (TIF) [file pgen.1010096.s004.tif]

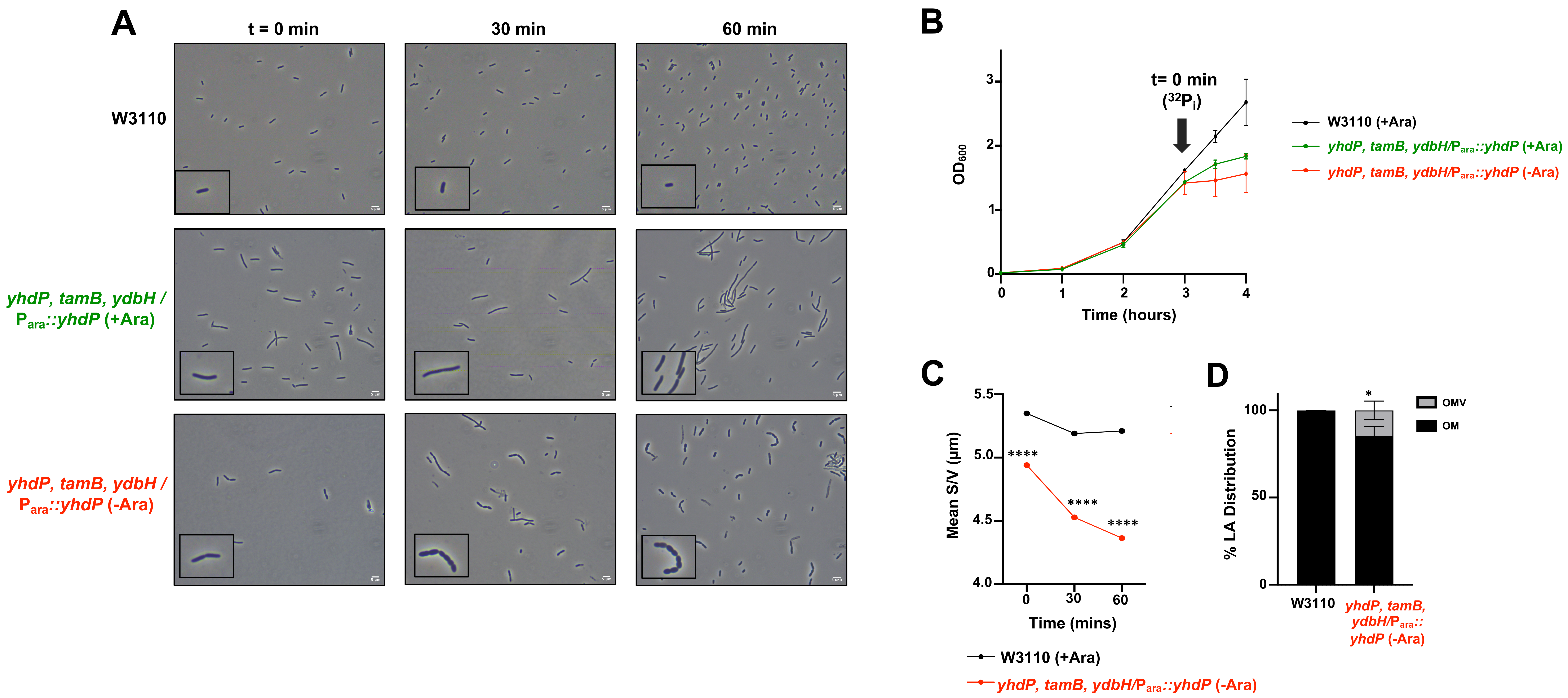

Supplement: S5 Fig — (A) Microscopy of yhdP, tamB, ydbH triple mutant expressing YhdP from an arabinose-inducible promoter (W3110 yhdP, tamB, ydbH/Para::yhdP). Phase contrast microscopy (1000x) of the indicated cells with white scale bar set to 5 μm. Image in bottom left corner of dividing cell is a 300x zoom of the larger image. Microscopy data is representative of two biological experiments. (B) Growth of yhdP, tamB, ydbH mutants. Strains were monitored by OD600 every hour at 37°C. After 3 hours of growth, cultures were inoculated with 32Pi and aliqouts were collected for further analysis. Error bars represent SD from biological triplicates. (C) Surface area/volume measurements of cells at beginning of 32Pi addition. Cell width and length of listed strains were measured using MicrobeJ software after strains were inoculated with 32Pi and imaged at 1000x on agarose pads, S/V was calculated using cell width and length. T-test used between strains. 0.0001>P****. (D) LPS distribution across OM and OMV. 60 minutes after W3110 and W3110 yhdP, tamB, ydbH/Para::yhdP grown in glucose was inoculated with 32Pi, lipid A was extracted from the collected OM and OMV fractions and lipid A distribution was calculated from TLC densitometry. T-test used between strains. 0.05>P*. (TIF) [file pgen.1010096.s005.tif]
