# Supplementary material for: Absence of YhdP, TamB, and YdbH leads to defects in glycerophospholipid transport and cell morphology in Gram-negative bacteria
Source: PLoS Genet. 2022 Feb 28;18(2):e1010096. doi: 10.1371/journal.pgen.1010096 (PMC8912898; doi:10.1371/journal.pgen.1010096)
Supplement: S1 Table — (DOCX) [file pgen.1010096.s006.docx]

**S1 Table: Strains, plasmids and primers used in this study**

**Strains**

| **Strain** | **Description** | **Source of reference** |
| --- | --- | --- |
| *E. coli* W3110 | Wild type, F- λ-, *rphH*-1 *IN*(*rrnD, rrnE*)1 | *E. coli* Genetic Stock Center (Yale) |
| WD101 | W3110, pmrA^C^ | (1) |
| MVD101 | WD101 Δ*yhdP::kan* | This study |
| MVD102 | WD101 Δ*yhdP,* derived from MVD101 | This study |
| MVD103 | WD101 Δ*yhdP* (pWSK29), derived from MVD102 | This study |
| MVD104 | WD101 Δ*yhdP* (pWSK29-*yhdP*), derived from MVD102 | This study |
| MVD113 | WD101 Δ*yhdP,* Δ*wzzE::kan* | This study |
| MVD114 | WD101 Δ*yhdP, lpxD_W192R_*, derived from MVD101 | This study |
| MVD115 | WD101 Δ*yhdP, lpxC_R41S_*, derived from MVD101 | This study |
| MVD116 | WD101 Δ*yhdP, lpxC_R41S_*, derived from MVD101 | This study |
| MVD117 | WD101 Δ*yhdP, lpxC_V236M_*, derived from MVD101 | This study |
| MVD118 | WD101 Δ*yhdP, lpxC_T76I_*, derived from MVD101 | This study |
| MVD119 | WD101 Δ*yhdP, lpxC_412IKETVR_*, derived from MVD101 | This study |
| MVD123 | WD101 Δ*asmA::kan* | This study |
| MVD124 | WD101 Δ*asmA,* derived from MVD123 | This study |
| MVD125 | WD101 Δ*tamB::kan* | This study |
| MVD126 | WD101 Δ*tamB,* derived from MVD125 | This study |
| MVD127 | WD101 Δ*ydbH::kan* | This study |
| MVD128 | WD101 Δ*ydbH*, derived from MVD127 | This study |
| MVD129 | WD101 Δ*yhjG::kan* | This study |
| MVD130 | WD101 Δ*yhjG,* derived from MVD129 | This study |
| MVD131 | WD101 Δ*yicH::kan* | This study |
| MVD132 | WD101 Δ*yicH*, derived from MVD131 | This study |
| MVD133 | WD101 Δ*yhdP,* Δ*asmA::kan,* derived from MVD102 | This study |
| MVD134 | WD101 Δ*yhdP,* Δ*ydbH::kan,* derived from MVD102 | This study |
| MVD135 | WD101 Δ*yhdP,* Δ*yhjG::kan,* derived from MVD102 | This study |
| MVD136 | WD101 Δ*yhdP,* Δ*yicH::kan,* derived from MVD102 | This study |
| MVD137 | WD101 Δ*yhdP* (pBAD-*yhdP*), derived from MVD102 | This study |
| MVD138 | WD101 Δ*yhdP,* Δ*tamB* (pBAD-*yhdP*), derived from MVD137 | This study |
| MVD139 | WD101 Δ*yhdP,* Δ*tamB::kan, lpxD_W192R_*, derived from MVD114 | This study |
| MVD143 | WD101 Δ*yhdP,* Δ*eptA,* Δ*tamB::kan,* derived from MVD142 | This study |
| MVD144 | W3110 Δ*yhdP::kan* | This study |
| MVD145 | W3110 Δ*yhdP,* derived from MVD144 | This study |
| MVD146 | W3110 Δ*tamB::kan* | This study |
| MVD147 | W3110 Δ*tamB,* derived from MVD146 | This study |
| MVD148 | W3110 Δ*yhdP,* Δ*tamB::kan,* derived from MVD145 | This study |
| MVD150 | W3110 *lpxC101* | This study |
| MVD151 | W3110 Δ*yhdP,* Δ*tamB,* derived from MVD148 | This study |
| MVD153 | W3110 Δ*yhdP,* Δ*tamB, lpxC101_,_* derived from MVD151 | This study |
| MVD155 | WD101 ΔyhdP*,* Δ*lpp::kan,* derived from MVD102 | This study |
| MVD156 | WD101 ΔyhdP*,* Δ*lpp,* derived from MVD155 | This study |
| MVD157 | WD101 ΔyhdP*,* Δ*lpp,* Δ*tamB,* derived from MVD156 | This study |
| MVD158 | W3110 Δ*yhdP,* Δ*tamB,* ΔydbH*/*P_ara_::*yhdP,* derived from MVD148 | This study |
| Plasmid | Description | Source of reference |
| pCP20 | FLP recombinase expression; Amp^R^ CamR; temperature-sensitive replicon | (2) |
| pKD4 | Plasmid containing a kan resistance cassette flanked by FRT sites | (3) |
| pKD46 | Plasmid which encodes a λ Red Recombinase system from temperature sensitive promoter | (3) |
| pBAD18 | High copy expression plasmid, Amp^R^, *P_ara_::* empty, PBR322 origin | (4) |
| pWSK29 | Low copy expression plasmid, *lac* promoter, Amp^R^ | (5) |
| pWSK29-*yhdP* | Amp^R^, *P_IPTG_::nativeRBS_yhdP* | This study |
| pBAD-*yhdP* | Amp^R^, *P_ara_::nativeRBS_yhdP* | This study |

**Primers**

| Primer name | DNA sequence (5’-3’)* |
| --- | --- |
| YhdP_F | gcagtttgacgtcgtaatgatg |
| YhdP_R | ccgacctacggttatgttctg |
| YhdP_NotI_RBS_F | taagacgcggccgctttagcagacaaggagtgacg |
| YhdP_XhoI_R | tgcttactcgaggcgcaacacttcgttgattt |
| YhdP_EcoRI_RBS_F | taagacgaattcttagcagacaaggagtgacg |
| YhdP_KpnI_R | gtcttaggtacctatgagattggggcaattacg |
| TamB_F | atagtggcgaagcggtaag |
| TamB_R | atagtggcccaggtataca |
| Lpp_F | aatccgatggaagcatcctg |
| Lpp_R | tatcgtcgggcagggtatc |

*Underlined sequences denote restriction enzyme cut sites

**Supplementary information references**

1. Trent MS, Ribeiro AA, Doerrler WT, Lin S, Cotter RJ, Raetz CRH. Accumulation of a Polyisoprene-linked Amino Sugar in Polymyxin-resistant Salmonella typhimurium and Escherichia coli STRUCTURAL CHARACTERIZATION AND TRANSFER TO LIPID A IN THE PERIPLASM. J Biol Chem. 2001 Nov 16;276(46):43132–44.

2. Cherepanov PP, Wackernagel W. Gene disruption in Escherichia coli: TcR and KmR cassettes with the option of Flp-catalyzed excision of the antibiotic-resistance determinant. Gene. 1995 May 26;158(1):9–14.

3. Datsenko KA, Wanner BL. One-step inactivation of chromosomal genes in Escherichia coli K-12 using PCR products. Proc Natl Acad Sci USA. 2000 Jun 6;97(12):6640–5.

4. Guzman LM, Belin D, Carson MJ, Beckwith J. Tight regulation, modulation, and high-level expression by vectors containing the arabinose PBAD promoter. Journal of bacteriology. 1995;177(14):4121–30.

5. Wang RF, Kushner SR. Construction of versatile low-copy-number vectors for cloning, sequencing and gene expression in Escherichia coli. Gene. 1991 Apr;100:195–9.
